# The Genetic Architecture of High Bone Mass

**DOI:** 10.3389/fendo.2020.595653

**Published:** 2020-10-29

**Authors:** Celia L. Gregson, Emma L. Duncan

**Affiliations:** ^1^Musculoskeletal Research Unit, Translational Health Sciences, Bristol Medical School, University of Bristol, Bristol, United Kingdom; ^2^Department of Twin Research & Genetic Epidemiology, School of Life Course Sciences, Faculty of Life Sciences and Medicine, King’s College London, London, United Kingdom

**Keywords:** high bone mass (HBM), osteopetrosis, *SOST*, *LRP5*, dual-energy X-ray absorptiometry (DXA), bone mineral density (BMD), genome-wide association studies (GWAS)

## Abstract

The phenotypic trait of high bone mass (HBM) is an excellent example of the nexus between common and rare disease genetics. HBM may arise from carriage of many ‘high bone mineral density [BMD]’-associated alleles, and certainly the genetic architecture of individuals with HBM is enriched with high BMD variants identified through genome-wide association studies of BMD. HBM may also arise as a monogenic skeletal disorder, due to abnormalities in bone formation, bone resorption, and/or bone turnover. Individuals with monogenic disorders of HBM usually, though not invariably, have other skeletal abnormalities (such as mandible enlargement) and thus are best regarded as having a skeletal dysplasia rather than just isolated high BMD. A binary etiological division of HBM into polygenic *vs.* monogenic, however, would be excessively simplistic: the phenotype of individuals carrying rare variants of large effect can still be modified by their common variant polygenic background, and by the environment. HBM disorders—whether predominantly polygenic or monogenic in origin—are not only interesting clinically and genetically: they provide insights into bone processes that can be exploited therapeutically, with benefits both for individuals with these rare bone disorders and importantly for the many people affected by the commonest bone disease worldwide—*i.e.*, osteoporosis. In this review we detail the genetic architecture of HBM; we provide a conceptual framework for considering HBM in the clinical context; and we discuss monogenic and polygenic causes of HBM with particular emphasis on anabolic causes of HBM.

## Introduction

Most people are first introduced to genetics through the gardening career of Gregor Mendel and his observations regarding various features of the pea plant (flower color, pod shape, etc.). Mendel’s studies led him to conclude that individual characteristics (*i.e.*, phenotypes) were determined by discrete units of information (*i.e.*, genes) that came in pairs (*i.e.*, alleles), with one of each pair inherited by each offspring randomly and independently of the genes determining other characteristics (1). He also concluded that at any particular locus one allele would be dominant and the other recessive.

Mendel’s laws certainly explained the phenotypes observed in his multigenerational plant breeding experiments; and they provided an explanation for the inheritance of autosomal monogenic disorders (2). However, they appeared not to explain the inheritance of many traits that exhibit continuous distribution in the population (*e.g.*, height, weight). Initial attempts at reconciliation proposed that continuously distributed phenotypes might still be determined by a single locus but with a ‘blending’ of each parent’s characteristics rather than a pure dominant/recessive model of inheritance (*e.g.*, a tall mother and a short father would have children of average height); but ultimately this question was resolved by the demonstration that continuously distributed (or quantitative) traits arise from the effect of multiple genetic loci, each of which individually exhibits Mendelian inheritance (3), which combine, both additively and interactively, and within a given environment, to produce the final phenotype.

These concepts are not just of historical interest but highly relevant when considering the genetic architecture of high bone mass (HBM)—or indeed any other heritable disease.

## What Is Genetic Architecture?

To quote Gratten et al., “genetic architecture refers to the number of genomic loci contributing to risk, the distribution of their allelic frequencies and effect sizes, and the interactions of alleles in and between genes, all of which contribute to the relationship between genotype and phenotype. Understanding genetic architecture is the foundation on which progress in dissecting etiology is built because it dictates which study designs for identifying risk variants are likely to be most successful.” (4) It is hard to improve upon this elegant definition and its clear consequences regarding gene mapping strategies [for an in-depth discussion of this topic, the reader is referred to an excellent recent review (5)].

In considering the genetic architecture of HBM specifically, the simplest question that can be asked is whether HBM is monogenic (due to carriage of a rare variant of large phenotypic effect) or polygenic (arising from the cumulative effect of multiple variants, each individually of small effect). However, even answering this apparently simple question is not straight-forward, as these are not necessarily mutually exclusive options, whether considering either the HBM population as a whole or a particular affected individual.

Monogenic diseases, whether dominant or recessive, autosomal or X-linked, are due to rare highly penetrant alleles affecting a single gene. Monogenic diseases generally follow classical Mendelian inheritance such that the presence or absence of disease is mathematically predictable, with some leeway for variable penetrance and expressivity from genetic and/or environmental modifiers (6). Although individually rare, the World Health Organization (WHO) estimates that monogenic disease affect 1% of the worldwide population (7); and there are many skeletal dysplasias that display classic Mendelian inheritance, with either high (*e.g.* osteopetroses) or low (*e.g.* osteogenesis imperfecta) bone mineral density (BMD) (8).

However, this does not mean that all heritable dichotomous disease states are monogenic. Many common diseases (*e.g.*, ankylosing spondylitis, osteoarthritis, breast cancer) are defined as present or absent according to particular characteristics, whereas other common diseases (*e.g.*, hypertension, type 2 diabetes) are defined using a threshold value along a continuously distributed phenotype (*i.e.*, blood pressure and glycemia). It is perhaps easier to understand how quantitative disease states may be polygenic in inheritance (3), compared with qualitative (*i.e.*, dichotomous) common disease states. However, qualitative diseases may also be polygenic: it is the underlying risk of disease that is quantitative, with disease manifest once a particular genetic threshold is reached (3, 9). Indeed, *a priori* even diseases that might appear monogenic are more likely to be polygenic (10). The validity of this concept has been demonstrated comprehensively by the enormous success of genome-wide association studies (GWAS), which have identified thousands of variants associated with a host of common quantitative and qualitative diseases as diverse as type 1 diabetes to schizophrenia to prostate cancer (11). The polygenic common variant ‘background’ can also modify the phenotype of persons carrying rare highly penetrant monogenic variants—such as *BRCA1* mutations in breast cancer or carriage of HLA-B27 in ankylosing spondylitis (12–14). Here, it is worth highlighting that extreme HBM populations are enriched with common variant ‘high BMD’ alleles (discussed further later in this article) (15).

In considering the translational applications of GWAS, it would be fair to say that at least initially the clinical utility of polygenic (or genomic) risk scores (PRS) calculated using genome-wide associated SNPs was underwhelming—certainly in bone disease. At the time of publication of the second GEnetic Factors in Osteoporosis Study [GEFOS-2], a study involving tens of thousands of cases and controls, the PRS derived from variants associated with femoral neck BMD at genome-wide significance (*i.e.*, p <5 × 10^−8^) performed less well in predicting BMD than age and weight alone (area under receiver-operator characteristics curve: 0.59 *vs.* 0.75) (16). This was not really surprising: despite the large sample size, the identified variants still explained only a small proportion (<6%) of overall BMD heritability (16). Over time, ever larger GWAS have been performed (17, 18); and certainly increasing GWAS population size strongly correlates (on a log scale) with the number of SNPs identified at genome-wide significance to be associated with disease (11), capturing a greater proportion of heritability, and improving PRS utility. Additionally, adopting a less stringent threshold for SNP inclusion in PRS also increases the proportion of genetic variance captured—at the cost of more noise and inclusion of more false-positive results. There is no fixed formula for the sweet spot between sensitivity and specificity for PRS (*i.e.*, maximizing AUCs in ROC analyses or similar statistic). It is disease-specific (19); and for maximal clinical utility the PRS must also be interpreted in the context of other disease-specific factors including disease heritability, prevalence, and prior probability (19–23). [For further discussion on the calculation and clinical utility of PRS, the reader is referred to two recent review articles (19, 23)]. However, despite these caveats, PRS have reached the point whereby, to quote Khera et al., “for a number of common diseases, polygenic risk scores can now identify a substantially larger fraction of the population than is found by rare monogenic mutations, at comparable or greater disease risk” (24). For example, at a population level, the proportion of individuals who carry a sufficient burden of common variants to place them at three-fold risk of coronary artery disease is twenty-fold that of individuals carrying rare highly penetrant LDL-R mutations of equivalent risk (24). Moreover, common variants can be easily and cheaply genotyped, without needing whole population whole-genome sequencing, noting that the choice of technology in this area is sometimes a political rather than a strictly scientific decision.

## Use of BMD to Define Dichotomous Disease States of Osteoporosis and High Bone Mass

Defining a disease state by use of a particular threshold value within the normal population distribution of a quantitative trait is a concept extremely familiar to the bone community. The most commonly employed measure of bone strength is BMD, usually assessed using dual-energy X-ray absorptiometry (DXA). The result is then compared against an age, ethnicity and sex-specific reference population, allowing calculation of T- and Z-scores (the number of standard deviations (SDs) by which the result differs from the mean BMD of a young adult or age-matched population, respectively). Individuals with lower BMD are at higher risk of fracture, particularly low trauma fractures (25). Reflecting this risk, in 1999 the WHO used DXA BMD to define osteoporosis and osteopenia (for osteoporosis, a T-score of ≤−2.5; for osteopenia, a T-score between −1 and −2.5) (26). These threshold definitions do not account for other major risk factors for fracture—such as age and prior fragility fracture, both of which independently increase future fracture risk (27)—and use of BMD in isolation to define the real clinical issue (*i.e.*, bone fragility and fracture risk) can lead to apparent paradoxes, *e.g.*, a woman with osteopenia (according to BMD) and a previous low trauma fracture is at higher risk of a fracture than a women with BMD-defined osteoporosis who has not yet fractured (28). Nevertheless, such thresholds are useful in identifying a high-risk group of clinical relevance, in whom intervention might be most clinically- and cost-effective. Further, fracture risk calculators [*e.g.*, FRAX, Garven (29)] have been developed to account for key clinical risk factors, as well as BMD, to circumvent the limitations of BMD alone.

At the other end of the normal distribution for BMD are individuals with HBM. It is tempting to regard these individuals simply as phenotypic outliers, with their BMD results of little serious clinical consequences unless such individuals unexpectedly find themselves in deep water (non-metaphorically). However, studying individuals with HBM is of relevance both for their own sake and for the community more broadly. Firstly, HBM may indicate an underlying and hitherto-unsuspected skeletal dysplasia with specific clinical needs (*e.g.*, monitoring of cranial nerve function, therapeutic choices, and genetic counseling) (30). Second, these individuals provide novel insights into the regulation of bone mass: such discoveries may inform not only therapeutic approaches to their own HBM condition but also for the opposite, and more prevalent, bone phenotype of osteoporosis. In considering this last point, an important caveat applies. While low BMD is closely related to increased fracture risk (25), the converse is not necessarily true (31). For example, individuals with high BMD due to disorders of bone resorption (*e.g.*, osteopetroses) or disturbed bone turnover (*e.g.*, Paget’s disease), can manifest high fracture rates.

## How High Is High Bone Density?

Epidemiological studies of high BMD are few and definition thresholds are variable (32, 33). Indeed, the absence of an upper limit to define ‘normal’ BMD risks those with a pathological cause for high BMD being missed and labeled as “normal.” In 2005, Michael Whyte proposed a high BMD definition of a Z-score >+2.5, to alert clinicians to this issue (30). However, until more recently publications around high BMD were still the purview of case reports and small case series.

To address this question, we conducted the first systematic analysis of patients undergoing routine clinical DXA scanning, encompassing 335,115 DXA scans across 15 UK centers. We first used a screening threshold T or Z-score ≥+4 at any lumbar/hip site to identify those with extreme high BMD, in whom we investigated the potential underlying causes for a high BMD, trying to identify within this heterogeneous population with high BMD, a sub-group with unexplained generalized HBM (identified using a Z-score threshold ≥+3.2) (discussed in detail later) (34). Overall, within this UK population, scanned by DXA over a retrospective 20 year period for a wide variety of more-or-less clinically justifiable indications, we found the prevalence a T or Z-score ≥+4 to be 0.5%, and within that of unexplained generalized HBM with Z-score ≥+3.2 to be 0.18% (34). Interestingly, reflecting on the mathematics of a normal distribution, four standard deviations (SDs) would be expected to identify just 0.003% of a population, while 3.2 SDs equates to 0.069% (35). Taken together, it seems BMD might have a marginally bimodal distribution at the upper tail of its distribution.

While this study was the first to assess the prevalence of high BMD within the general population, the—albeit large—population composed of individuals referred for DXA scanning for clinical reasons, rather than selected to represent the general population. Thus, selection bias is possible. However, as most individuals are referred for DXA due to a pre-test suspicion of low BMD and/or osteoporosis (*e.g.*, a history of steroid use), if anything the true prevalence of high BMD may have been underestimated to date. Thus, this study provides a minimal prevalence for this condition.

## Detecting High BMD in Clinical Practice

Incidental high BMD results in clinical practice are relatively common (34), and we have previously published an approach to guide their assessment and investigation (36). The commonest causes for a high BMD are artefactual, with osteoarthritic degeneration explaining half of all high BMD measurements (34) (see **Table 1** for list of artefacts). Importantly, identifying the presence of artefact in someone with apparently high BMD on DXA does not mean fracture risk is necessarily low; and artefact is important to recognize as it may mask osteoporosis. For example, an osteoporotic vertebral fracture with vertebral collapse will reduce measured bone area while maintaining bone mineral content, and thus increase calculated BMD.

**Table 1:** Causes of a high BMD measurement on a DXA scan.

Artefactual causes of raised BMD—no true increase in bone mass			Genetic Contribution		Refs.
			Monogenic	Polygenic	
Osteoarthritis			^a^	Yes	(34, 37)
DISH: Diffuse idiopathic skeletal hyperostosis			Yes	Yes	(38–41)
Ankylosing spondylitis				Yes^b^	(42, 43)
Vertebral fractures			Yes^b^	Yes	(17, 34)
Vascular calcification			Yes	Yes	(44–48)
Thalassemia major			Yes		(49, 50)
Gaucher’s disease (splenomegaly overlies the lumbar spine DXA field)			Yes		(34, 51)
Abdominal abscesses					(52)
Gallstones					(53, 54)
Renal calculi			Yes		(54, 55)
Gluteal silicon implants					(56)
Intestinal barium					
Surgical metalwork					(34)
Laminectomy					(57)
Vertebroplasty & kyphoplasty					
**Acquired causes of true increased bone mass and/or density**					
**Localised**	Tumors	Primary malignancies*e.g. osteoblastoma, Ewing’s sarcoma, carcoinoid, hemangioma, plasmocytoma, Hodgkin*’*s disease*Secondary osteosclerotic metastases*e.g. prostate, breast, gastric, colonic, cervical carcinoma*	Yes	Yes	(58)

	Chronic infective osteomyelitis				
	SAPHO (Synovitis Acne Pustulosis Hyperostosis and Osteitis) syndrome			Yes	(59–61)
	CKD-MBD (Chronic Kidney Disease-Metabolic Bone Disorder)^c^		Yes	Yes	(62–64)
	Paget’s disease of Bone (PDB)		Yes	Yes	(34, 65, 66)
	Early onset Paget’s like syndromes		Yes		(67)
	X-linked hypophosphatemia (XLH)		Yes		(68)
	Osteogenesis imperfecta associated with mutations affecting the carboxy-terminal-propeptide cleavage site of the type 1 procollagen chain		Yes		(69)
	Gnathodiaphyseal dysplasia		Yes		(70, 71)
**Generalized**	Fluorosis				(72–74),
	Acromegaly		Yes	Yes	(75, 76)
	Hepatitis C-associated osteosclerosis				(77–79),
	Myelofibrosis		Yes	Yes	(80–82),
	Mastocytosis		Yes		(83–85),
	Oestrogen replacement implants				(86)

a While there are no forms of monogenic OA, there are many monogenic skeletal dysplasias with degenerative joint disease—e.g. spondyloepiphyseal dysplasia tarda, achondroplasias.

b vertebral fractures occur in osteogenesis imperfecta.

c CKD-MBD increases in BMD can also be generalized.

Interestingly, many artefactual causes of high BMD are themselves heritable. The most common example is spinal osteoarthritis with osteophytosis (**Table 1**). The heritability of osteoarthritis is approximately 50%, and two large GWAS published in the last two years have identified association with 96 loci (37, 87). As another example, ankylosing spondylitis [AS] is highly (>90%) heritable (88), and associated with over 100 loci, in addition to HLA-B27 (42). AS artefactually elevates BMD through syndesmophyte formation at vertebral margins, anterior longitudinal ligament ossification, and scoliosis (43). It is also associated with increased fracture risk (89), which may be due to the rigidity of the axial skeleton, the presence of inflammation, or a combination of both. A further example is diffuse idiopathic skeletal hyperostosis (DISH), most commonly seen in older men and characterized by widespread spinal calcification (38), which is also heritable (39), though as yet no causative variants have been published (90). The relationship between DISH and abnormal phosphate handling, may also carry implications for bone mineralization, bone strength, and fracture risk, though this has not been formally assessed. The closely related disease ossification of the posterior longitudinal ligament (OPLL) is also heritable (91), with both common and rare susceptibility variants identified (92, 93)—though as this condition most commonly affects the cervical spine, a site not routinely screened by DXA, OPLL is less likely to cause clinical conundrum as an artefactual cause of high BMD in daily practice.

Calcification of structures anterior to the spine but within the DXA field can artefactually elevate BMD measurements (**Table 1**). Although vascular calcification of the abdominal aorta is common, reported in 43% of patients having lumbar DXA assessment (mean age 68 years), it is surprising how little evidence there is regarding the effect of this on lumbar spine BMD measures (94–97). The relationship between vascular calcification and low BMD is of particular interest (98), most evident (though not exclusively) in the chronic kidney disease population. Abdominal aortic calcification is associated with lower BMD and vertebral fractures (99); and the extent to which genetic pleiotropy underpins vascular calcification [itself heritable (44)] and osteoporosis is the source of active investigation. There are also monogenic forms of vascular calcification (for example, pathogenic variants in *ABCC8* causing pseudoxanthoma elasticum (MIM264800) [45–47)].

Beyond artefact, there are a number of other conditions, usually acquired through life, that cause true increases in bone mass and density, which may be localized or generalized.

## Localized Increases in BMD

From a clinical perspective, the most important question when faced with a localized increase in BMD is whether this might represent a tumor. Tumors causing local increases in BMD may be benign or malignant, primary or secondary (**Table 1**); in this context special mention must be made of breast and prostate cancer, both of which are associated with osteosclerotic bony metastases.

Paget’s disease of the bone (PDB) explains 1.4% of incidental high BMD results (34)—though this figure may fall given the declining population prevalence of PDB (current UK age-adjusted prevalence of 2.5% and 1.6% for men and women respectively) (100). Excessive and disorganized woven and lamellar bone expands bone size and raises density, causing focal increases in BMD but also increasing deformity and risk of fracture. PDB commonly affects the lumbar spine and hips [after the pelvis, the commonest sites of involvement are lower lumbar vertebrae (101)] and may be monostotic (*e.g.*, affecting an isolated vertebra) or polyostotic. PDB is often asymptomatic and may be present for years before diagnosis. PDB also displays both monogenic and polygenic inheritance. Mutations in *SQSTM1* (p62) account for 40% of familial and 10% of sporadic PDB (MIM167250) (65); and other monogenic forms of PDB include the more severe and/or early onset PDB caused by mutations in *ZNF687*, *FKBP5*, and *TNFRSF11A* (which codes for RANK). Common variants in loci harboring the genes *CSF1*, *OPTN*, *TM7SF4*, and *RIN3* have also been implicated (65). It is thought that environmental triggers interact with this genetic architecture to predispose to disease, with one hypothesized environmental trigger being zoonotic infections (102).

In additional to classical PDB, a number of rarer Paget’s-like syndromes have been described with onset early in life that can also cause localized increases in measured BMD. These include expansile skeletal hyperphosphatasia, familial expansile osteolysis (FEO) (MIM174810), Juvenile Paget’s disease (MIM239000), early-onset familial Paget’s disease (MIM602080), and panostotic expansile bone disease (67). Children present with deafness, dental disorders and on occasion, active focal bone lesions; and as in PDB alkaline phosphatase levels tend to be raised. These conditions are due to genetic mutations in the RANK-NFkappaB signaling pathway [comprehensively reviewed in (103)].

Mutations affecting the carboxy-terminal-propeptide cleavage site of the type 1 procollagen chain (*COL1A1*) cause an unusual form of osteogenesis imperfecta in which individuals manifest marked bone fragility while having high BMD, due to hyperosteoidosis and hypermineralization. Patchy sclerotic lesions are often evident in the spine and elsewhere; in particular, these individuals develop unusual fibro-osseous lesions in the jaw (“cementoma”) (69). There is a clinical overlap of this condition with gnathodiaphyseal dysplasia (70), which features also include bone fragility, irregular sclerotic BMD, and fibro-osseus lesions in the skull and jaw. Gnathodiaphyseal dysplasia is associated with mutations in *ANO5 (*71*)*, a gene not known to be involved in collagen production or processing; and the overlap in phenotype between these conditions is not fully understood. However, recent studies have suggested that ANO5 may be involved in osteoclast regulation (104).

SAPHO syndrome (Synovitis, Acne, Pustulosis, Hyperostosis and Osteitis) is a rare and poorly understood condition, in which about half the cases manifest spinal involvement including patchy osteosclerosis, hyperostosis, and para-vertebral ossification (59, 60). Clustering within families is reported and a genetic etiology (including an HLA contribution) has been suggested (61).

Chronic kidney disease-mineral bone disorder (CKD-MBD, previously referred to as renal osteodystrophy) causes osteomalacia, secondary hyperparathyroidism, and fracture. Radiological features of CKD-MBD include bony sclerosis, particularly of the vertebral body endplates, leading to a ‘rugger-jersey’ spine appearance (an appearance distinctive for hyperparathyroidism); or it can be more diffuse (62–64). CKD-MBD is associated with markedly increased fracture risk (64).

## Generalized High BMD

A number of causes of generalized high BMD may be acquired through life (**Table 1**). For example, fluoride causes diffuse axial osteosclerosis with ligamentous calcification, periostitis and vertebral osteophytosis, and has been associated with excessive tea and toothpaste consumption (72–74). The increase in BMD led to fluoride being historically trialed as an osteoporosis therapy—but it resulted in a higher fracture risk, emphasizing that high BMD *per se* does not necessarily equate to stronger bones (105, 106). Other rare acquired causes of generalized high BMD are listed in **Table 1**.

However, rarer still, but fascinating are the monogenic causes of generalized high bone density, known as high bone mass (HBM) syndromes; these we discuss next.

## Monogenic Causes of Generalized High BMD

Several rare genetic disorders with skeletal effects, collectively termed osteopetroses and sclerosing bone dysplasias, are associated with generalized increased BMD. The most recent (10^th^) edition of the Nosology and Classification of Genetic Skeletal Disorders (2019 revision) lists 462 genetic disorders of the skeleton among which are 45 conditions characterized by osteosclerosis or osteopetrosis, with the underlying gene(s) identified in 40 conditions at the time of going to press (8). As suggested previously (36), and per a recent review paper of de Ridder et al. (107), an intuitive biological separation can be made into disorders in which bone formation is enhanced, those in which bone resorption is depressed, and those with a disturbed balance between bone formation and resorption. Importantly, the associated changes in bone structure and quantity in the various sclerosing bone disorders can have quite different—indeed, completely opposite—effects on fracture risk (8).

It is not our intention to discuss all 45 osteosclerotic and osteopetrotic conditions listed in the current edition of the Nosology (8). Rather, we will focus on cases illustrative of the differences between types of monogenic high BMD, with a particular focus on anabolic HBM.

## Genetic Causes of Increased Bone Formation and High BMD

A common feature of anabolic HBM is activation of the Wnt/β-catenin signaling pathway, with increased signaling through this pathway underlying the phenotype of sclerosteosis, van Buchem’s disease, *LRP4* HBM, *LRP5* HBM, and *LRP6* HBM (all discussed below). For a detailed discussion of Wnt signaling in bone, the reader is referred to the excellent review of Baron and Kneissel (108). A brief—and, acknowledged, simplistic—description of the canonical Wnt/β-catenin signaling pathway is provided here. Wnt ligands bind to the dual receptor complex comprising Frizzled and LRP5 or LPR6 [LRP5/6], resulting in β-catenin escaping phosphorylation by being released from a multiprotein β-catenin “destruction complex”, leading to β-catenin accumulation in the cytoplasm and ultimately translocation to the nucleus to activate target genes. In the absence of Wnt binding, β-catenin is phosphorylated by GSK-3β (a component of the “destruction complex”) leading to its degradation and, consequently, loss of downstream signaling. Sclerostin inhibits Wnt signaling, by binding to LRP5/6 and preventing LRP5/6 from forming the dual receptor complex with Frizzled. LRP4 anchors sclerostin, enhancing sclerostin’s interaction with LRP5/6, thus facilitating sclerostin’s inhibition of Wnt/β-catenin signaling (108, 109).

**Figure 1:**
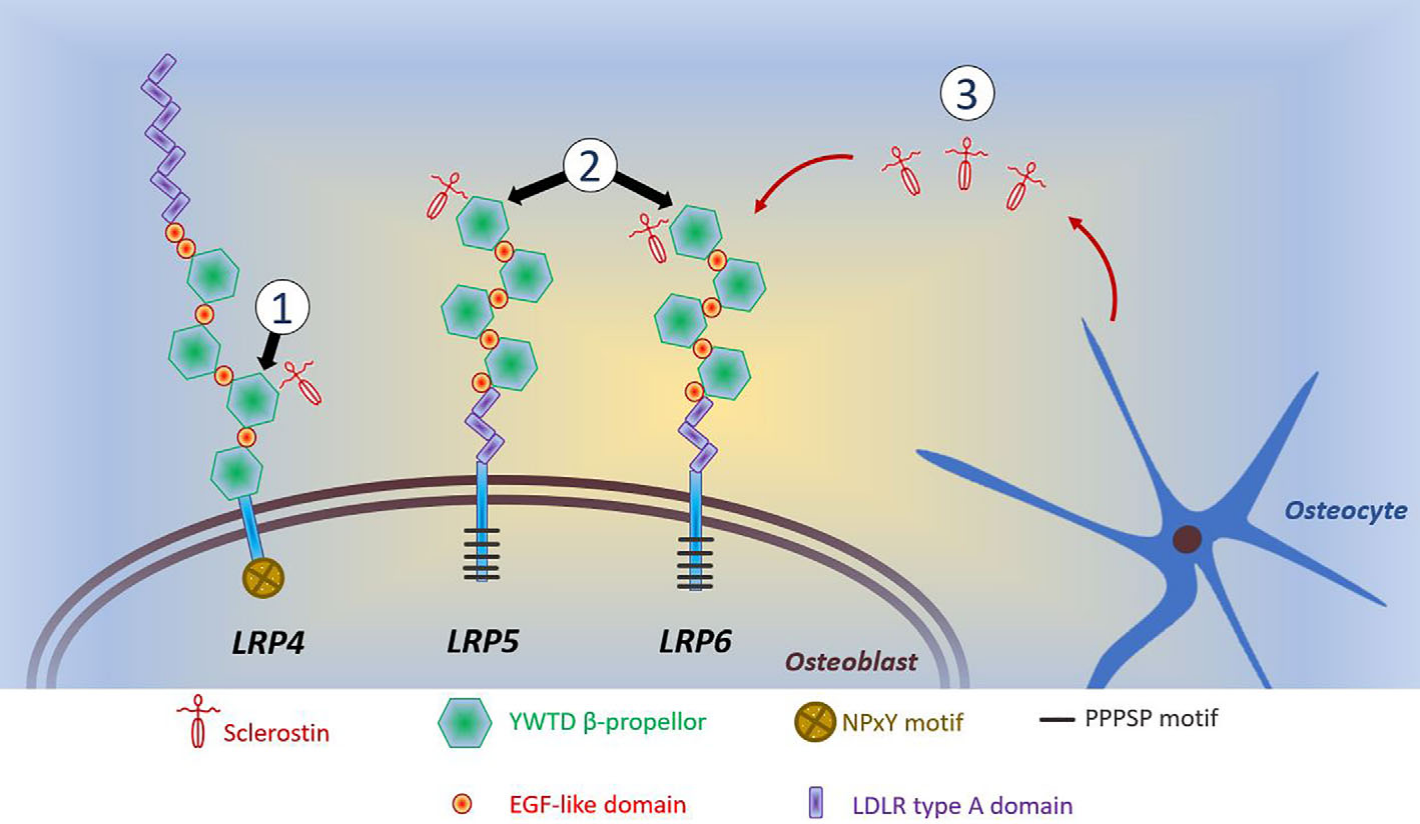
Schematic diagram of reported mutations affecting osteoblastic Wnt signaling. (1) *LRP4* mutations coding for the 3^rd^ β-propellor impair sclerostin binding; (2) *LRP5* and *LRP6* mutations coding for the 1^st^ β-propellor impair sclerostin binding; (3) *SOST* mutations inhibit sclerostin production by osteocytes. Reductions in the inhibitory effects of sclerostin allows LRP5/6 to interact with Wnt and its co-receptor Frizzled, which prevents phosphorylation of β-catenin allowing it to accumulate in the cytoplasm of the osteoblast. Translocation of β-catenin to the nucleus activates transcription of target genes. This activation of canonical Wnt/β-catenin signaling increases osteoblastic bone formation. The intracellular consequences of LRP4-sclerostin binding are less well characterized; however, reductions in LRP4-sclerostin binding have a similar effect to increase osteoblastic bone formation. LDLR, low-density-lipoprotein receptor. LRP, LDLR related proteins; PPPSP, Proline, Proline, Proline, Serine, Proline; EGF, epidermal growth factor; NPxY, Aspartate, Proline, any amino acid, Tyrosine; YWTD, Tyrosine, Tryptophan, Threonine, Aspartate.

Several human diseases characterized by HBM are associated with mutations of components of the Wnt/β-catenin signaling pathway (see **Figure 1** and text below). As a corollary, mutations of other components of this pathway may also cause HBM, with several such examples evident from mouse genetic studies (108). Thus, sequencing efforts in human populations may lead to the identification of other anabolic HBM conditions in humans.

### Sclerosteosis and van Buchem’s Disease

Sclerosteosis (MIM269500) and van Buchem’s disease (MIM239100) are rare, clinically similar conditions of excessive bone growth. Loss-of-function *SOST* mutations cause sclerosteosis, generally thought the more severe of the two disorders; in contrast, a 52-kb intronic deletion downstream of *SOST*, thought to disrupt post-transcriptional sclerostin processing, results in the milder phenotype of van Buchem’s disease (110, 111). In both disorders, reduced osteocytic production of sclerostin permits activation of osteoblastic Wnt signaling, leading to enhanced bone formation, increased bone strength, and resistance to fracture (110, 112) (**Table 2**). Understanding the molecular biology of sclerosteosis and van Buchem’s disease has led to the development of monoclonal antibodies against sclerostin, which act to suppress the inhibitory action of sclerostin on Wnt signaling, allowing gains in bone formation (147, 148). Thus, anti-sclerostin antibodies represent a new class of anti-osteoporosis therapy; and recently the first-in-class agent (romosozumab) was approved by the United States Food and Drug Administration and the European Medicine Agency (discussed further below).

**Table 2:** Inherited HBM conditions due to enhanced bone formation: gene defects, function, and clinical characteristics.

Condition	MIM	Inheritance	Gene	Mutation	Protein	Function	Clinical Features	Ref
Increased bone formation								
**Sclerosteosis**	269500	AR	*SOST*	Loss of function	Sclerostin	Osteoblast Wnt signaling inhibitor	Cutaneous digital syndactyly excessive height. Skull/mandible thickening, tori**^a^**, CN palsies (incl. neonatal),. Headaches, raised ICP, coning. Back/bone pain. Fracture resistance	(110, 113–115)
**Van Buchem’s Disease****^b^**	239100	AR	*SOST**^c^*	Reduced function	Sclerostin	Osteoblast Wnt signaling inhibitor	No syndactyly, no excess height. Skull/mandible thickening, tori**^a^**, CN palsies. Headaches, back/bone pain. Fracture resistance	(110, 116, 117)
***LRP4* HBM**	604270	AD & AR	*LRP4*	Loss of function	LRP4	Impaired sclerostin-LRP4 interaction	Syndactyly, dysplastic nails, gait disturbance, facial nerve palsy, deafness	(118, 119)
***LRP5* HBM**	603506	AD	*LRP5*	Gain of function	LRP5	Osteoblast cell membrane co-receptor regulating Wnt signaling	Asymptomatic or tori**^a^**, skull/mandible thickening, CN palsies, neuropathy, neuralgia, headaches, back/bone pain, spinal stenosis, reduced buoyancy, craniosyntosis, increased height. Fracture resistance	(120–139),
***LRP6* HBM**	awaited	AD	*LRP6*	Gain of function	LRP6	Osteoblast cell membrane co-receptor regulating Wnt signaling	Mandible thickening, torus palatinus, teeth encased in bone, absence of adult maxillary lateral incisors, inability to float. Fracture resistance. Increased height	(138)
***SMAD9* HBM**	awaited	AD	*SMAD9*	Loss of function	SMAD9	Inhibits BMP dependent targetgene transcription to reduce osteoblast activity	Mandible enlargement, broad frame, torus palitinus/mandibularis, pes planus, increased shoe size, inability to float	(140)
**Cranio-metaphyseal dysplasia**	123000218400	AD	*ANKH*	Gain of function	Homolog of mouse ANK	Osteoclast-reactive vacuolar proton pump	Macrocephaly, cranial hyperostosis CN palsies, wide nasal bridge, dental overcrowding, craniofacial hyperostosis & sclerosis, metaphyseal flaring, and high BMD	(141–143),
		AR	*GJA1*	Loss of function	Gap junction protein alph‐1			
**Lenz‐Majewski hyperostotic dysplasia**	151050	SP	*PTDSS1*	Gain of function	Phosphatidylserine synthase 1	Phospholipid biosynthesis	Mandible enlargement, generalized hyperostosis, proximal symphalangism, syndactyly, brachydactyly, cutis laxa, developmental delay, hip dislocation, marked hypertelorism, and enamel hypoplasia	(144, 145)

MIM^®^, Online Mendelian Inheritance in Man; CN, Cranial Nerve; ICP, Intracranial pressure; BMP, bone morphogenetic protein.

a Tori: Oral exostoses which include torus palatinus & mandibularis; found in approximately 25% of a general Caucasian population (146).

b Initially known as hyperostosis corticalis generalisata familiaris (116, 117).

c A 52-kb intronic deletion downstream of SOST.

Sclerosteosis causes a large skeleton (sometimes termed ‘gigantism’, though this term is more usually reserved for children with excess long bone growth due to growth hormone excess prior to epiphyseal closure), mandible enlargement, and torus palatinus and mandibularis which can complicate tooth extractions (113, 149). Calvarial overgrowth compresses cranial nerves, particularly facial nerves, sometimes from infancy; in one series 83% of 63 adults had recurrent facial nerve palsies (113). Hearing loss and headaches are common; as is raised intracranial pressure—to the point that craniotomy may be required to prevent sudden death by coning (113, 150). Cutaneous syndactyly of fingers (present in 76%) and toes is an important defining feature, often accompanying dysplastic or absent nails and camptodactaly (113, 150, 151). Sclerosteosis is progressive, which may cause bone and back pain, with bony overgrowth requiring spinal and cranial decompression (113).

Van Buchem’s disease is milder than sclerosteosis, importantly without syndactyly or “gigantism” (110, 150); however, cranial nerve impingements and hearing loss remain common (152). Management is generally limited to surgical bone removal. However, as tried in Camurati-Engelmann disease (progressive diaphyseal dysplasia, MIM131300) glucocorticoids have been used with the aim of reducing high bone turnover, in an isolated case report (153).

### *LRP5* High Bone Mass

In 1997, a family with HBM but an otherwise normal phenotype was reported with the genetic abnormality localized by linkage analysis to chromosome 11q12–13 (120). The 18-year-old proband had presented following a road traffic accident, without bone injury, but with consequent back pain. In the initial publication, 28 family members were phenotyped, aged 18 to 86 years. Inheritance of the HBM phenotype was autosomal dominant. Affected individuals were asymptomatic and had never fractured; their biochemistry was normal (measured in a subset of 5 affected individuals); and their radiology showed dense bones with thick cortices and reduced medullary cavity but without consequent reduction in hemopoietic capacity. Affected individuals had spinal BMD Z-scores ranging from approximately +3.2 to +7.9; authors used a case definition threshold of Z-score > +3.0. They concluded that as HBM affected individuals aged as young as 18 years of age, the mutation has a role to play in the acquisition of peak bone mass; similarly, the clinical evaluation of older members of the pedigree supported a persistent influence throughout life without consequent disability (120, 121).

Interestingly, around the same time, osteoporosis pseudoglioma syndrome (OPPG) (MIM259770), was mapped to the same region (154). OPPG is characterized by osteoporosis, extreme bone fragility, fracture, and deformity; and although initially considered autosomal recessive, obligate carrier parents usually have low BMD. OPPG is due to inactivating mutations in *LRP5* (155). OPPG also leads to visual deterioration at birth or soon after, due to vitreoretinal degeneration, with multiple consequences including retrolental masses, retinal detachment, cataract, phthisis bulbi, microphthalmia, vitreous hemorrhage, secondary glaucoma and blindness (156). Inactivating *LRP5* mutations have also been associated with familial exudative vitreoretinopathy type 4 (FEVR-4) (MIM601813), with low BMD a common feature of affected individuals also (157); and it is now recognized that FEVR-4 and OPPG are allelic disorders with overlapping phenotypes. Notably, other forms of familial exudative retinopathy are associated with mutations in other genes that affect Wnt signaling, including *LRP4* and *FZD4*.

Further genetic analysis of the original HBM family, with extension of the pedigree to 38 members, identified a mis-sense *LRP5* mutation (c.512G>T, p.Gly171Val), in exon 3. All affected individuals were heterozygous for this mutation, consistent with autosomal dominant inheritance (121). HBM affection status in this kindred-based study was then defined as sum of hip and spine Z-score > +4.

In contrast to inactivating *LRP5* mutations associated with OPPG, the HBM phenotype results from activating *LRP5* mutations which stimulate osteoblastic bone formation (158). *LRP5* has 23 exons, coding for a 1615 amino acid protein, an essential cell membrane co-receptor key to the Wnt signaling pathway which regulates osteoblastic bone formation (122). The majority of the protein constitutes the extracellular β-propellor which has four domains (1180 amino acids in length). All HBM-associated *LRP5* mutations identified to date lie in exons 2, 3 and 4, which collectively code for the 1st β-propellor domain (**Figure 1**; **Table 3**); and protein modeling suggests they all lie at the top/central region of the extracellular protein (163). It is thought that these mutations reduce binding affinity with sclerostin and Dkk1, negative regulators of LRP5 signaling (163, 164). No inactivating mutations in this 1st β-propellor domain have been identified to date; instead, OPPG-associated mutations have been located within the 2nd and 3rd β-propellor domains, the binding domain, and the terminal signaling peptide (123, 165, 166). A hallmark of increased Wnt signaling in many organs, other than bone, is the development of malignant tumors (167, 168). However, fortunately this has not been reported as a feature of *LRP5* HBM.

**Table 3:** *LRP5* mutations and associated clinical and radiological characteristics reported to date.

No. of reported individuals^a^	*LRP5* base change	Amino acid change	Exon	Country	Ethnicity	Tori TP & TM	Mandible	Neurological complications	Fracture History (#)	Clinical Features other than HBM (reported in at least one person in the kindred)	Radiology	Biochemistry	Refs
1	*c.266A>G*	p.Gln89Arg	2	UK	Caucasian	No	No	Carpel tunnel syndrome	None	Osteoarthritis	NDG	NDG	(159)
1	*c.331G>T*	p.Asp111Tyr	2	Argentina	NDG	NDG	Enlarged mandible. Mandibular pain	Headaches	NDG	Severe headaches, extremity pain	Dense cranium, loss of diploe, enlarged mandible, increased cortical thickness of long bones	NDG	(127)
6 of 15	*c.461G>T*	p.Arg154Met	2	USA from Lithuania	Caucasian	Yes^b^	Enlarged mandible	None	None	Pain in right hip after prolonged standing in the index case	Increased density of calvarium, mandible & endosteal surface of long bones	Calcium, PO_4_, bALP normal	(123)
2 of 2	*c.509_514dupGGGGTG*	p.G171_E172insGG	3	Austria	NDG	No	NDG	Congenital deafness, VII palsy	None	Cochlear implant, migraine. Removal of occipital bone in one individual. Hypergonadotropic hypogonadism.	Increased calvarial and cortical thickness. Foramen magnum stenosis	Osteocalcin normal. CTX normal in mother, raised in daughter	(160)
3	*c.511G>C*	p.Gly171Arg	3	Belgium	NDG	NDG	NDG	Headaches	NDG	Severe headaches in one affected individual	Dense skull bones, cortical thickening of the vertebrae and long bones with normal development	NDG	(127)
1	*c.511_516delGGTGAG*	g.69547_69552delGGTGAG	3	NDG	Caucasian	NDG	Thickened mandible	Hearing impairment. Sudden sight loss aged 16	None	Generalized bone pain, headaches	Calvarial thickening. Restriction of auditory and optic canals	Calcium, PO_4,_ ALP normal. PTH mildly raised.	(161)
19 of 38	*c.512G>T*	p.Gly171Val	3	USA	Caucasian	NDG	NDG	None	Resistant to #	Asymptomatic	All bones of skeleton radiologically dense, thick cortices, reduced medullary cavity, normal shape	bALP, osteocalcin, deoxy- & pyridinoline X-links normal in subgroup of 5 affected	(120, 121)
7 of 16	*c.512G>T*	p.Gly171Val	3	Connecti-cutUSA	Caucasian	Yes	Wide, deep mandible,decreased mandibular angle	None	None	Asymptomatic other than difficulty floating	Thickened mandibular rami, marked cortical thickening of long bones, dense vertebrae but shape normal	bALP Ca, PO_4_, PTH, OPG, RANKL, & urinary NTX normal.Osteocalcin, elevated. All in subgroup of 4 affected	(124)
1	*c.512G>T*	p.Gly171Val	3	Colorado USA	NDG	Yes	Wide deep mandible	Strabismus, Bells’ palsy, trigeminal neuralgia, headaches, paraesthesias	NDG	Bone painPseudotumor cerebri, type 1 Chiari malformation	Dense skeleton, marked thickening of skull, and skull base, cortical widening that narrowed medullary cavity of the long bones	bALP and osteocalcin normal	(125)
6 of 13	*c.512G>T*	p.Gly171Val	3	NDG	NDG	TP in all but 1 case	Wide deep mandible	Deafness, sensorimotor neuropathy, dysphonia, spinal stenosis	None	One affected individual required surgery for spinal stenosis, another underwent hip replacement, with difficult surgery attributed to unusual hardness of bone	NDG	NDG	(126)
2 of 2	*c.512G>T*	p.Gly171Val	3	NDG	NDG	Yes	Wide deep mandible	No detail given	None	One individual had hydromyelia (a complication of type 1 Chiari malformation)	NDG	NDG	(126)
1	*c.518C>T*	p.Thr173Met	3	UK	Caucasian	No	No	Ulna nerve decompression	2 high impact	OsteoarthritisPalpable enthesophyte at tibial tubercle	NDG	NDG	(159)
1 of 4	*c.592A>T*	p.Asn198Tyr	3	NDG	NDG	Yes	NDG	No	None	HBM despite steroids. Ear canal decompression surgery. Headaches, back pain	Severe cortical thickening of cranial and long bones	P1NP increased. Calcium, PO_4,_ ALP, PTH normal	(162)
2 of 6	*c.593A>G*	p.Asn198Ser	3	NDG	NDG	Yes	Wide deep mandible	Deafness, sensorimotor neuropathy, & spinal stenosis	None	NDG	NDG	NDG	(126)
3	*c.593A>G*	p.Asn198Ser	3	UK	Afro-Caribbean	Yes	Enlarged mandible	None	None	Individuals affected differently Chest wall prominence Fixed flexion at elbows Osteotomy of tibial tubercle (for tendonitis)..:. HBM despite steroids	Increased calvarial thickness	Low bone turnover on Alendronic acid	(159)
?	*c.640G>A*	p.Ala214Thr	3	Portland USA	NDG	Yes	Elongated mandible	NDG	Resistant to #	Similar phenotype to that described by Boyden et al., 2002.	Increased density of calvarium, mandible and endosteal surface of long bones	NDG	(127, 134)
13 of 24	*c.640G>A*	p.Ala214Thr	3	Holland	NDG	None	Prominent mandible	CN VII palsy in 2 cases	None	Craniosynostosis, developmental delay, tinnitus, headaches, prominent forehead present in >1 member of pedigree	Increased density of calvarium, mandible, pelvis & endosteal surface of long bones	Calcium, PO_4_, bALP normal	(129)
1	*c.641C>T*	p.Ala214Val	3	UK	NDG	NDG	Enlarged mandible	NDG	NDG	Similar phenotype to that described by Boyden et al., 2002	Similar to Boyden et al., 2002.	NDG	(127, 130)
Family 1:1 of 1Family 2 & 3 unknown	*c.724G>A*	p.Ala242Thr	4	Portland USA, & Sardinia	NDG	Yes	Enlarged mandible	None in one case (393). No detail given in 2 families	Resistant to #	NDG	Increased density of calvarium. Mandible and endosteal surfaces of long bones	NDG	(127, 133–135)
?	*c.724G>A*	p.Ala242Thr	4	France	NDG	Yes	Enlarged mandible	NDG	Resistant to #	Osteomyelitis of the jaw, hearing difficulties due to small auditory canals in 2 affected individuals	Increased density of calvarium, enlargement of cranial vault	NDG	(136)
2 of 10	*c.724G>A*	p.Ala242Thr	4	UK	Caucasian	Yes	Enlarged mandible	None	None	Renal calculi (in one individual)Dental overcrowding	NDG	Increased bone turnover at age 21	(159)
1	*c.724G>A*	p.Ala242Thr	4	UK	Caucasian	Yes	Enlarged mandible	CN V & VII mildly impaired	None	Widespread arthralgia, shin pain & headaches	Increased calvarial thickness with tightly packed brain gyri on MRI. Anterior lumbar syndesmophytes	NDG	(159)
2	*c.724G>A*	p.Ala242Thr	4	UK	Caucasian	Yes	Enlarged mandible	Conductive deafness	None	Osteoarthritis	NDG	NDG	(159)
Family 1:13 of 32Family 2:7 of 16	*c.758C>T*	p.Thr253Ile	4	Fyn,Denmark	NDG	NDG	NDG	NDG	No increased # rate	NDG	Generalized sclerosis including calvarium (obliteration of frontal sinuses & mastoids), pelvis & long bones.Enlargement of cranial vault	NDG	(127, 128, 131, 132)
1	*c.796C>T*	p.Arg266Cys	4	UK	Caucasian	Yes	Enlarged mandible	None	None	HBM despite steroids	NDG	Normal bone turnover	(159)
1	*c.844A>G*	p.Met282Val	4	Belgium	NDG	Yes	None	None	NDG	Knee pain, chondrocalcinosis & OA. Cervical spine pain. Developed breast cancer	Thickened skull and long bones on MRI and phalanges on X-ray. Increased density of vertebral bodies without OA	Calcium, PO_4_, bALP, CTX all normal	(137)

# fracture CN Cranial nerve; TP, torus palitinus; TM, torus mandibularis; bALP, bone specific alkaline phosphatise; PO_4_, phosphate; PTH, parathyroid hormone; CTX, NTX, C and N-telopeptides cross-links of bone Type I collagen; UK, United Kingdom; NDG, No detail given.

a No. of reported individuals (with pedigree size where reported).

b 3 requiring surgical debulking of TP & TM.

Comprehensive description of the clinical phenotype of *LRP5* HBM was reported by Boyden et al., in 2002 (124). Seven individuals from a family of 16 were analyzed after routine DXA screening detected two related individuals with high BMD, again due to *LRP5* c.512G>T, p.Gly171Val. Again, radiographic thickening of cortices of otherwise normal bones was reported, without history of fracture. However, all cases had deep wide mandibles and torus palitinus. Two of 7 cases reported difficulty floating; the first-time buoyancy was reported in association with bone density in humans. Serum calcium, phosphate, ALP, nf-kB and osteoprotegerin (OPG) were normal, as was urinary NTX-1. However, unlike the first family, osteocalcin was elevated threefold (mean 32.3 (SD 7.4) *vs.* 9.8 (1.8) ng/mL). This finding was suggestive of a stimulatory effect on periosteal bone formation, leading to an increase in cortical thickness, as has been previously reported in transgenic mice expressing the p.Gly171Val mutation (169).

While Boyden et al.’s large family were asymptomatic of their HBM trait, in a letter of response two years later Whyte et al. reported a 37 year old woman with the same pathogenic variant (*LRP5* c.512G>T, p.Gly171Val) but a more severe phenotype, including congenital strabismus, childhood Bells’ palsy, trigeminal neuralgia, life-long headaches, bone pain, and paresthesias (125). She also had a widened and enlarged mandible; and her osseous tori had required dental intervention and removal as they had encased her teeth from an early age. Interestingly, this more extreme case had a normal osteocalcin level.

In their letter of response Boyden et al. detailed a further three unrelated families with *LRP5* HBM, two with the same *LRP5* c.512G>T, p.Gly171Val variant and one with a novel variant in *LRP5* c.593A>G (p.Asn198Ser), also in exon 3 (126) (**Table 3**). Among these families, some affected individuals had also reported deafness, sensorimotor neuropathy and spinal stenosis, all likely due to bone overgrowth and nerve compression. Contemporaneously, van Wesenbeeck et al. identified a further six novel *LRP5* mutations spread through exons 2, 3 and 4 (127). Cases generally had similar features of mandible enlargement, fracture resistance, increased skull thickness and oral tori.

Sixteen activating *LRP5* mutations affecting 29 families have now been reported globally, all in exons 2, 3 and 4 and affecting the 1st β-propeller domain (**Table 3**) (120, 121, 123–137, 159–162). Almost all are missense mutations, with two indels reported. Half of all cases report osseous tori (see below); and only one case has experienced fractures (notably, after high impact). As increasing numbers of individuals are reported, it is apparent that *LRP5* HBM may not be as benign as first thought, and indeed shares many features with sclerosteosis and van Buchem’s disease (**Table 2**)—which is not particularly surprising given they share a common pathway. In addition to the adverse clinical features reported by Whyte et al. (detailed above) (125), Kwee et al. reported a multi-generation family with an *LRP5* c.640G>A, p.Ala214Thr mutation, in whom the phenotype extended beyond HBM to include craniosynostosis, developmental delay, and multiple dysmorphic features including macrocephaly and hypertelorism (129). Premature closure of cranial sutures in one infant reportedly caused raised intracranial pressure, optic nerve atrophy and visual impairment (this child also manifest a ventriculoseptal defect); two other individuals had required craniotomy. Foramen magnum stenosis, in one case ultimately requiring craniotomy, has also been reported in association with the only HBM-associated *LRP5* insertion reported to date (c.509_514dupGGGGTG, p.G171_E172insGG) (160). The p.Gly171Val mutation has been reported twice in association with a type 1 Chiari malformation (125, 126). Headaches and bone pain are common; bony compression of the optic and auditory nerves causing loss of sight and hearing respectively are frequently reported; and mandibular osteomyelitis, renal calculi and spinal stenosis have also been reported (126, 136, 159). However, joint disease does not appear to be a common feature, with osteoarthritis reported only in some older individuals (159).

Certainly, there will be a bias toward identification of more severe HBM phenotypes associated with *LRP5* mutations, as these are more likely to be identified clinically and thus reported. In our own study mentioned earlier, in which we screened 335,115 DXA scans across 15 UK centers, we identified seven families with *LRP5* HBM; two with novel and five with previously reported mutations (159). The two novel mutations had milder phenotypes than those reported previously, and arguably would have been less likely to have been identified without gene screening. Our findings suggested that the clinical variability in *LRP5* HBM cases may arise from genotype-phenotype correlation, with our protein modeling suggesting the severity of high BMD corresponds to the degree of predicted LRP5 protein disruption (159).

### *LRP4* High Bone Mass

To date, four cases of HBM associated with pathogenic variants in *LRP4* have been published, with both autosomal dominant and recessive inheritance reported (109, 118, 119, 170, 171). Mutations causing *LRP4* HBM occur in the central cavity of the third β-propeller domain of the LRP4 protein, impairing the interaction between sclerostin and LRP4 (119) (of note mutations elsewhere in *LRP4* are associated with Cenani-Lenz Syndactyly Syndrome, MIM212780) (**Figure 1**). In addition to HBM, clinical features of *LRP4* HBM include syndactyly, dysplastic nails, gait disturbance, facial nerve palsy and hearing loss (of note, no osseous tori have been reported to date) (**Table 2**) (119). As the phenotype of *LRP4* HBM is very similar to sclerosteosis, it has been termed sclerosteosis type 2 (118)—though the clinical course is less severe and arguably more similar to van Buchem’s disease.

### *LRP6* High Bone Mass

In 2019 Whyte et al. reported two multi-generational families with *LRP6*-associated HBM, identifying two different heterozygous missense mutations both affecting the first β-propeller of LRP6 (homologous to *LRP5* HBM mutations) (**Figure 1**) (138).

The clinical features of *LRP6* HBM are highly reminiscent of *LRP5* HBM: generalized osteosclerosis and hyperostosis, mandible enlargement, torus palitinus, teeth encased in excessive bone, resistance to fracture, and an inability to float in water (**Table 2**), with an additional phenotypic feature of absence of adult maxillary lateral incisors in some individuals (observed in both families). Of note, no signs of osteoarthritis were detected.

Interestingly, not only was *LRP6* HBM associated with above-average height, this paper also highlighted increased height in individuals with *LRP5* HBM (who were studied for comparison with the *LRP6* families) (138). Taken together, the phenotypes suggest increased Wnt signaling seen in all three conditions affects not only bone density but also skeletal growth in childhood and adolescence.

### Other Forms of High BMD With Increased Bone Formation, Not Associated With Wnt Signaling Pathways

#### *SMAD9* High Bone Mass

In 2019 we reported the first pedigree with a segregating *SMAD9* mutation, with replication in two further unrelated individuals with HBM. Based our population size (34), we estimated the prevalence of *SMAD9* HBM as approximately 1 in 100,000, less common than *LRP5* HBM (159). As with *LRP5* HBM, the clinical phenotype included mandible enlargement, a broad frame and tall stature, torus palatinus, and a tendency to sink when swimming; and no adult fractures were reported (**Table 2**). A further characteristic, not reported in *LRP4, LRP5*, or *LRP6* HBM, was pes planus. Reassuringly, unlike sclerosteosis and some cases of *LRP5* HBM, nerve compression was not seen (159).

*SMAD9* (also known as *SMAD8, MADH6*, and *MADH9*) encodes a downstream modulator of the BMP signaling pathway. BMPs are members of the TGF-β superfamily, and induce both bone and cartilage formation (172). Our *in-silico* protein modeling predicted the mutation severely disrupted the structure of the MH1 DNA binding domain of *SMAD9*, leading to loss-of-function, such that this inhibitory SMAD could no longer repress BMP receptor activation and downstream signaling (173). Our novel findings support the SMAD9-dependent BMP signaling pathway as a potential novel anabolic target for future osteoporosis therapeutics.

#### Craniometaphyseal Dysplasia

Craniometaphyseal dysplasia (MIM123000), which may be autosomal dominant or recessive, is caused by a mis-sense mutation in *ANKH*, which encodes the inorganic pyrophosphate channel ANK. The phenotype includes macrocephaly, cranio-facial hyperostosis and sclerosis with cranial nerve palsies, wide nasal bridge, dental overcrowding, metaphyseal flaring and marked HBM (the latter predominantly in AR disease) (141–143).

#### Lenz‐Majewski Hyperostotic Dysplasia

Autosomal dominant gain of function mutations in *PTDSS1* are responsible for Lenz–Majewski syndrome (LMS) (MIM 151050) (174). This very rare syndrome is characterized cutis laxa, facial dysmorphism, severe short stature, brachydactyly, intellectual disability and hyperostotic skeletal dysplasia. Skeletal characteristics include calvarial thickening, marked sclerosis of the skull base and facial bones, a markedly enlarged mandible (much more so than is seen in the Wnt signaling HBM syndromes), dense vertebral bodies, shortened broad ribs, hyperostotic clavicles, scapulae and iliac wings (144, 145). Progressive osteosclerosis with “massive thickening of long tubular bones” is described by the age of 30 years (144). Bilateral hip dislocation has been reported. *PTDSS1* codes for phosphatidylserine synthase 1 (PSS1), an enzyme involved in phospholipid biosynthesis, although the mechanism by which this affects bone metabolism is not yet fully understood (174).

#### Osseous Tori

Oral exostoses include torus palatinus (TP), torus manibularis (TM) and, less commonly, torus maxillaris. Their site determines their nomenclature (with TP lying in the midline of the hard palate and TM usually in the premolar region of the lingual side of the mandible). The size and number of tori an individual may have is highly variable. They are each made up of dense cancellous bone with a surrounding rim of cortical bone; occasionally they contain hemopoietic tissue (175). The only apparent clinical problem associated with tori *per se* is obstruction to dentition (including denture fitting); and tori rarely require surgical de-bulking. Notably, tori are not present in all cases of *LRP5 or LRP6* HBM (**Table 3**).

Although prevalence estimates have varied widely (between 1 and 64% depending upon the study, definition and population), overall tori appear to be relatively common (approximately 25% of a Caucasian population, clearly much higher than the prevalence of HBM) and appears similar across all ages (146). Interestingly, two separate studies (one among US (90% Caucasian) postmenopausal women and another in elderly Japanese women) have found an association between tori and higher BMD (176, 177). The US study graded tori size (0 to 5) and found a strong correlation with BMD Z-score among 469 women; however, they did not find a similar correlation between age and torus size (176).

Taken together, these data suggesting that torus may reflect acquisition of peak bone mass in early adult life rather than a progressive skeletal change. Moreover, tori do not appear to be sensitive or specific indicators of a monogenic form of HBM but may simply reflect a general association with higher peak bone mass.

## Unexplained HBM—A New Entity?

Mutations in the genes mentioned above are extremely rare within the general population, and the vast majority of HBM cases (~97%) remain genetically unexplained (159). Based on our UK study, *LRP5* HBM mutations have an estimated prevalence of approximately 5 per 100,000 (159). We identified only one sclerosteosis carrier, who manifested moderately high BMD due to a novel heterozygous nonsense *SOST* mutation predicted to either prematurely truncate sclerostin or cause nonsense-mediated decay (159). No cases of autosomal recessive sclerosteosis, *LRP4* HBM or *LRP6* HBM have been identified in the UK to date (159).

Thus there remains a population with generalized raised BMD (Z-score ≥+3.2 at either L1 or hip), usually identified incidentally on routine DXA scanning (34, 159), in whom fracture risk is not increased, with clinical characteristics suggestive of a mild skeletal dysplasia (associated features of mandible enlargement, extra bone at the site of tendon and ligament insertions, broad skeletal frame and larger shoe size, poor buoyancy, as well as an increased BMI) (34). The population is characterized by increased trabecular BMD and by alterations in cortical bone density and structure, leading to substantial increments in predicted cortical bone strength (178). Neither trabecular nor cortical BMD appear to decline with age in the tibia of HBM individuals, suggesting resistance to age-related bone loss in weight-bearing limbs may contribute to their bone phenotype (178). Furthermore, body composition assessment suggests that HBM is associated with a marked increase in fat mass, particularly android fat, in women but not men (179). This clinical appearance of a mild skeletal dysplasia explains 35% of incidental identified high BMD on routine DXA scanning, such that unexplained generalized HBM has a prevalence of 0.18% among a UK DXA-scanned adult population (34).

Within our cohort with unexplained HBM, 41% have a first-degree relative with a similar phenotype; thus unexplained HBM appears to be heritable though this figure has not been formally calculated (34). As mentioned above, mutations of other components of the Wnt/β-catenin pathway have been associated with HBM in murine genetic studies; and it may be these HBM individuals carry rare variants in genes yet to be identified through further sequencing efforts. However, this population with unexplained HBM is enriched for ‘high BMD alleles’ of loci identified through BMD GWAS in the general population. Thus, the genetic architecture of unexplained HBM is, at least in part, explained by common variants (15, 16). This does not exclude the possibility of rare variants of large effect in other genes in some (or all) of this cohort; rather, the effect of such variants with large effect may be modified by their background polygenic architecture.

As higher (*i.e.*, non-artefactually elevated) BMD is associated with prevalent osteoarthritis in the general population (180–183), it is perhaps not surprising that individuals with unexplained HBM have a greater prevalence of radiographic osteoarthritis than their unaffected family members and general population controls, along with a higher incidence of joint replacement (184–186). Interesting, when assessing the individual radiographic sub-phenotypes of osteoarthritis, be it at the hip, knee or hand, osteophytes predominate, with some increased subchondral sclerosis, rather than joint space narrowing (185–187). Taken together this suggests HBM might be associated with a hypertrophic ‘bone-forming’ osteoarthritis phenotype (188). While increased adiposity is also a clinical feature of HBM (with weight a major contribution to the development of degenerative joint disease), there remains an association between BMD and osteophytes, even after adjusting for BMI, at both weight-bearing (*e.g.* knee) and non-weight-bearing (*e.g.* distal interphalangeal [DIP] and carpometacarpal [CMC] joints of the hand) joints (186, 187).

More recently, we have been able to follow-up a proportion of our original HBM cohort eight years after initial assessment. We have observed increases in knee osteophytes and joint space narrowing, as well as knee pain and functional limitation (189); findings at the hip are similar (A Hartley et al., submitted for publication). Taken together, these insights from the study of an extreme HBM population suggest that raised BMD may contribute to pathogenesis of osteoarthritis.

## Osteopetroses and Osteosclerotic Conditions With Disturbed Formation and Resorption

### High BMD due to Osteoclast Dysfunction

The osteopetroses (Greek etymology: “petro”—to turn to stone) are rare genetic conditions of reduced osteoclastic bone resorption. Defective bone remodeling during growth induces skeletal sclerosis and abnormally dense but brittle bones. First described by the German radiologist Albers-Schönberg as “marble bone disease,” (190, 191) osteopetrosis is now classified by clinical severity (**Table 4**). The worst prognosis is seen in severe neonatal or infantile forms; a number of intermediate forms have been identified; and later-onset forms characterise the other end of the clinical spectrum (8, 237). Autosomal dominant osteopetrosis (ADO) was historically subdivided into ADO type I and type II. However, ADO type I was subsequently identified as high bone mass due to *LRP5* (low-density lipoprotein receptor-related protein 5) mutations (128) (discussed earlier). As *LRP5* HBM is not primarily a disease of osteoclasts, and is not characterized by bone fragility, we agree with the most recent edition of the Nosology [compared with the 2015 Nosology (237)] that *LRP5* HBM should not be considered an osteopetroses. *LRP5* HBM has now been reclassified within the group of “other sclerosing bone dysplasias” (8).

**Table 4:** Osteopetrotic conditions and osteosclerotic conditions with disturbed formation and resorption.

Condition	MIM	Inheritance*		Gene	Mutation	Protein	Function	Symptoms	Ref
**Severe/neonatal/infantile. autosomal recessive osteopetrosis****^a^**	259700604592	AR(OPTB1)		*TCIRG1*	Loss of function	T-cell, immune regulator 1, H+ transporting, lysosomal subunit A3 of V-ATPase pump	Acidification of the resorption lacuna	Fractures, infections (*e.g.* osteomyelitis), macrocephaly, frontal bossing, neurologic symptoms, CN compression, blindness, deafness, delayed tooth eruption, hemopoietic failure, death (usually before aged 10)	(190, 192, 193)
	602727611490	AR (OPTB4)		*CLCN7*	Loss of function	Chloride Channel	Acidification of the resorption lacuna		
	259720 607649	AR (OPTB5)		*OSTM1*	Loss of function	Osteopetrosis associated transmembrane protein 1	β-subunit for CLC-7		
	615085	AR (OPTB8)		*SNX10*	Loss of function	Sorting Nexin 10	Acidification of the resorption lacuna	Macrocephaly, broad open fontanelle, frontal bossing, small chin, and splenomegaly, severe optic atrophy with blindness, anemia, thrombocytopenia	(194, 195)
	602642259710	AR (OPTB2)		*RANKL/TNFSF11*	Loss of function	Receptor Activator for Nuclear Factor κ B ligand/tumor necrosis factor (ligand) superfamily, member 11	Osteoclastogenesis, resorption, survival	Osteoclast poor osteopetrosis. Fractures, hydrocephalus, nystagmus, seizures, hypersplenism, less severe course than *TCIRG1*, *CLCN7*, *OSTN1, SNX10* mutations	(196)
	603499612302	AR (OPTB7)		*RANK/TNFRSF11A*	Loss of function	Receptor Activator for Nuclear Factor κ B^b^	Osteoclastogenesis, resorption, survival
**Intermediate autosomal recessive osteopetrosis**	259710	AR (OPTA2)		*CLCN7*	Partial loss of function	Chloride Channel	Acidification of the resorption lacuna	Onset in childhood, fractures, short stature, cranial nerve compression	(192, 197)
	259700, 611497	AR (OPTB6)		*PLEKHM1*	Loss of function	Pleckstrin homology domain containing family M (with RUN domain), member 1	Vesicular trafficking	Osteopetrosis of the skull only (L2-L4 T-score -2.3). Fractures. Raised osteocalcin	(198)
**Osteopetrosis with renal tubular acidosis**	259730, 611492	AR (OPTB3)		*CA2*	Loss of function	Carbonic anhydrase II	Intracellular acidification	Developmental delay, short stature, CN compression, blindness, dental complications, fractures, maintained hemopoietic function.	(190, 192)
**Osteopetrosis with ectodermal dysplasia and immune defect**	300301	XL (OLEDAID)		*IKBKG*	Loss of function	Inhibitor of kappa light polypeptide gene enhancer in B-cells kinase gamma (NEMO),	Unknown	Lymphoedema, severe infections, no teeth, skin abnormalities, early death	(193)
**Leucocyte adhesion deficiency syndrome and osteopetrosis**	612840	AR (LAD-3)	*KIND3/FERMT3*		Loss of function	Kindlin-3/Fermitin-3	Cell adhesion	Bacterial infections, bleeding, osteopetrosis, hepatosplenomegaly	(199)
	612840	AR	*CalDAG-GEF1/RASGRP2*		Loss of function	Calcium and diaclyglycerol-regulated guanine nucleotide exchange factor 1			(200)
**Osteosclerotic metaphyseal dysplasia (OSMD)**	615198	AR	*LRRK1*		Loss of function	Leucine-rich repeat kinase 1	Osteoclast function; sealing zone formation	Developmental delay, seizures, metaphyseal osteosclerosis, diaphyseal osteopenia of long bones. Recurrent fractures. Skull unaffected.	(201, 202)
**Late onset osteopetrosis (Albers-Schönberg disease) ADOII**	166600	AD (OPTA2)	*CLCN7*		Dominant negative effect	Chloride Channel	Acidification of the resorption lacuna	Classic radiographic features, fractures, nerve compression, osteomyelitis, dental complications.	(192, 203–206)
**Pycnodysostosis**	265800, 601105	AR	*CTSK*		Loss of function	Cathepsin K	Collagen degradation	Delayed cranial suture closure, short stature and phalanges, dental abnormalities, fractures	(207–209)
**Osteopoikilosis**	166700	AD	*LEMD3*		Loss of function	LEM domain-containing 3	Disrupted BMP and TGFβ signaling pathways	Benign incidental osteosclerotic foci (can mimic metastases),^c^	(192, 210–214)
**Melorheostosis**	155950	AD	*LEMD3/MAP2K1SMAD3*		Loss of function	LEM domain-containing 3Mitogen-Activated Protein Kinase Kinase 1SMAD Family Member 3		Characteristic radiographic features asymmetric ‘flowing hyperostosis’ or ‘dripping candle wax’. Soft tissue changes (hypertrichosis, fibromas, hemangiomas and pain): associated with radiographic features in sclerotome. Contractures can develop
**Osteopathia striata**^d^ **with cranial stenosis**	300373	XL (OSCS)	*WTX/AMER1*		Loss of function	Wilms tumor gene on the X chromosome/APC Membrane Recruitment Protein 1	Wnt signaling suppression	Macrocephaly, CN compression, cleft palate, skull/long bone sclerosis in females. Usually lethal in males	(162, 215)
**Dysosteosclerosis**	224300	AR	*SLC29A3CSF1R*		Loss of function	Solute carrier family 29 (nucleoside transporter)Colony Stimulating Factor 1 Receptor	Osteoclast differentiation and function	Neurodevelopmental deterioration, platyspondyly, cranial nerve compression, abnormal dentition	(216–219)
**Diaphyseal dysplasia Camurati-Engelmann**^e^	131300	AD	*TGFβ1*		Probable gain of function	TGFβ	Cell proliferation, differentiation, migration and apoptosis	Variable phenotype. Thickened diaphyseal cortices, limb pain, fatigability, muscle weakness, waddling gait. Variably raised ALP, hypocalcemia & anemia	(220–226)
**Ghosal hematodiaphyseal syndrome**	274180	AR	*TBXAS1*		Loss of function	Thromboxane synthase	Modulates RANKL & OPG expression	Impaired platelet aggregation (steroid-sensitive), anemia. Similar to Camurati-Engelmann syndrome but metaphyses also involved	(227, 228)
**Trichodentoosseous dysplasia**	190320	AD	*DLX3*		Loss of function	Distal‐less homeobox 3	Ectodermal development	Sparse curly hair, severe dental abnormalities, defective tooth enamel. Sclerosis of calvaria and/or long bones	(229)

MIM^®^ Online Mendelian Inheritance in Man CN, Cranial Nerve; RANKL, Receptor Activator of Nuclear Factor-_K_β Ligand; OPG, Osteoprotegerin; XL, X-linked; ADOII, Autosomal dominant type 2 osteopetrosis.

a ARO incidence is 1/200,000–300,000 live births (193).

b As well as an osteoclast poor ARO phenotype, RANK mutations have also been linked to the Paget’s-like diseases (familial expansile osteolysis, expansile skeletal hyperphosphatasia and early-onset Paget’s disease); (230, 231)

c When associated with connective tissue naevi, dermatofibrosis lenticularis disseminata then termed Buschke-Ollendorff syndrome (192, 210, 232)

d can occur in combination with focal dermal hypoplasia, skin pigmentation, hypoplastic teeth, syndactyly, ocular defects and fat herniation through skin and is known as Goltz Syndrome (233–236).

e Also known as progressive diaphyseal dysplasia.

### Osteopetrosis, Late‐Onset Form Type 2 (OPTA2), Previously Known as Autosomal Dominant Osteopetrosis II (ADOII)

OPTA2 (MIM166600, eponymously known as Albers-Schönberg disease) is caused by *CLCN7* mutations. CLCN7 functions as a voltage-gated Cl-/H+ ion channel, and is found in lysosomes and on the ruffled boarder of osteoclasts. By acid efflux, it facilitates inorganic bone matrix dissolution (238). Mutations in *CLCN7*, therefore, result in decreased osteoclastic bone resorption. Multiple mutations have been identified throughout the gene, in association with a range of osteopetrotic phenotypes (239–241). The prevalence of OPTA2 is estimated between 0.2 and 5.5/100,000 (242, 243); however, it exhibits both variable penetrance (60–80%) and expressivity, results in a varied clinical phenotype including detection as an incidental radiographic finding (244). The phenotype can include facial nerve palsy, visual loss (in 5–25%), carpal tunnel syndrome, hip osteoarthritis (in 7%), increased fracture risk and delayed fracture healing, osteomyelitis (in 10–13%, particularly in the mandible), dental abscesses (10%) and deep decay (36%) and, in extreme cases, bone marrow failure (≈3%) (192, 203–206).

Radiographs feature (a) vertebral end-plate thickening (another cause of ‘rugger-jersey spine’), (b) ‘bone-within-bone’ particularly in the pelvis, and (c) transverse sclerotic bands within the distal femorae (203, 206). However, the radiological phenotype is not ubiquitous (≈60–90%) (233, 242). DXA BMD Z-score ranges from +3 to +15 (203, 205).

OPTA2 highlights that high BMD does not necessarily equate to lower fracture risk. In one case series of 94 *CLCN7* mutation cases, almost every adult (98%) had experienced a fracture (including, in half of carriers, their hip), with a third having fractured more than once (five had >15 fractures) (205). Among another 42 cases from 10 families, age range 7 to 70 years, the mean number of fractures per person was 4.4 (205). However, these case series are not performed systematically; thus, patterns are difficult to generalise.

### Pycnodysostosis

First described in 1962 and said to be the malady of both Toulouse-Lautrec and Aesop (known for his fables) (234–236), pycnodysostosis (MIM265800) is caused by defective enzymatic degradation of organic bone matrix, due to an autosomal recessive mutation in *CATK* (coding for cathepsin K) (207). To date approximately 30 mutations have been reported among fewer than 200 cases globally (207–209). Secreted by osteoclasts, cathepsin K cleaves type I collagen (245). The characteristic bone dysplasia includes skull deformities, under-developed facial bones with micrognathia, beaked nose, short stature and phalanges, dental caries, persistence of deciduous teeth and abnormally dense but brittle bones (192, 207–209, 246). Affected individuals may also manifest hip fractures indistinguishable clinically from atypical femoral fractures associated with antiresorptive therapy (247). Interestingly, particularly in light of the previous statement, the molecular understanding of pycnodysostosis underpinned development of a novel class of anti-resorptive therapy (248), although ultimately this agent did not make it to market (see below).

### β3-Integrin Disorders Associated With Platelet Dysfunction and Osteopetrosis

β3-integrins act with filamentous actin to facilitate podosome attachment of osteoclasts to bone. β3-integrin double knock-out mice develop osteosclerosis, with increased cortical and trabecular mass, as well as hypocalcemia, due to defective osteoclast function (249). *ITGB3* encodes glycoprotein IIIa which is the β subunit of the glycoprotein IIb/IIIa cell adhesion complex. Interestingly this IIb/IIIa complex acts as a fibrinogen receptor and mediates platelet aggregation; it is this complex which the widely used cardiological drugs tirofiban and abciximab target in their anti-platelet action as glycoprotein IIb/IIIa inhibitors, used at the time of percutaneous coronary interventions. Hence unsurprisingly, dysfunction of β-integrins appears to cause defective platelet aggregation and HBM in mice models (250). Autosomal recessive mutations in *ITGA2B* lead to reduced production of either glycoprotein IIb or IIIa, resulting in Glanzmann thrombasthenia (MIM273800) which is characterized by excessive bleeding (251). Only one case of Glanzmann thrombasthenia has been reported with a bone phenotype, with generalized and skull base osteosclerosis observed on plain radiographs of a 5 day old baby (252), termed osteopetrosis and thought due to impaired osteoclast function (253). A similar platelet phenotype has been associated with osteopetrosis (reported in three cases) in the presence of mutations in *Kindlin-3* (MIM612840), coding for Kindlin-3 which also interacts with β-integrins. The resulting condition is termed leukocyte adhesion deficiency-3 (LED-3) and predisposes to bacterial infections and bleeding despite normal platelet counts, as well as a bony phenotype (199).

## Other Osteosclerotic Disorders

### Osteopoikilosis and Melorheostosis

Osteopoikilosis (MIM166700; Greek etymology: poikilos- various) is benign, usually incidental finding characterized radiographically by multiple small round osteosclerotic foci, which can cause concern for metastases. When associated with connective tissue naevi, (dermatofibrosis lenticularis disseminate) it is termed Buschke-Ollendorff syndrome (BOS) (192, 210, 232). Melorheostosis, an asymmetric radiographic appearance of ‘flowing hyperostosis’ described as ‘dripping candle wax’ down the bone, can co-occur with osteopoikilosis. Approximately 200 cases have been described to date. Soft tissue signs and symptoms (see below) are associated with the radiographic features in a sclerotome distribution. Hypertrichosis, fibromas, hemangiomas and pain are sometimes a feature; and contractures and deformity can develop if limbs can become unequal in length (192, 210–212).

### Osteopathia Striata

Osteopathia striata can occur in combination with cranial sclerosis (MIM300373) or focal dermal hypoplasia (known as Goltz Syndrome; MIM305600)); both are X-linked dominant diseases and cause striations visible on bone radiographs, together with learning difficulties. In the former, which is due to mutations in *AMER1*, cranial osteosclerosis can lead to cranial nerve compression (215). In Goltz syndrome, caused by mutations in *PORCN*, the bone features are associated with skin pigmentation, hypoplastic teeth, syndactyly, ocular defects, and fat herniation through skin (254–256).

### Camurati-Engelmann Disease

Camurati-Engelmann disease (progressive diaphyseal dysplasia) (MIM131300) results from a gain-of-function mutation in *Transforming Growth Factor Beta-1* (*TGFB1*), resulting in thickened diaphyseal cortices, increased BMD, limb pain, fatigability, muscle weakness and a waddling gait (220). TGF-β controls cell differentiation, proliferation and apoptosis in many tissues; and its pivotal role in bone regulation is highlighted by the number of skeletal diseases associated with abnormal TGF-β signaling, which include Marfan’s syndrome, Loey-Dietz syndrome, acromesomelic and geleophysic dysplasias and even osteogenesis imperfecta (257).

### Ghosal Syndrome

Ghosal syndrome (MIM231095) is a rare autosomal recessive disorder caused by a mutation in *TBXAS1*, which encodes thromboxane synthase, resulting in HBM, impaired platelet aggregation, and anemia. The phenotype is not dissimilar from Camurati-Engelmann disease; however, here metaphyses are also affected (227, 228). This condition has linked platelet function with the RANKL/OPG pathway *in vitro* as thromboxane synthase modulates both RANKL and OPG expression in osteoblasts (228).

### X-Linked Hypophosphatemia

X-linked hypophosphatemia (XLH) (MIM307800), caused by phosphate-regulating endopeptidase homolog (*PHEX*) mutations, has also been reported as a cause of modestly elevated axial, though not appendicular, BMD, in both children (258) and adults (68). However, given the high prevalence of ligamentous calcification and degenerative joint disease in adults with XLH, interpreting a DXA BMD result is complex. Individuals with XLH have a high prevalence of pseudo- and complete fractures, with mean age at first fracture of 26 years (259). However, pertinent to the point regarding fracture *vs.* BMD, these fractures typically affect the lower limbs, noting that appendicular BMD is not usually increased in XLH; and are usually attributed to the combination of osteomalacia and mechanical stress (from rickets and joint mal-alignment).

### Neonatal Osteosclerotic Dysplasias

A handful of rare mutations can cause osteosclerosis in the neonate. Caffey disease (MIM114000), also known as infantile cortical hyperostosis, is a highly unusual bone disease causing excessive bone overgrowth (two-three times normal width—to the point of bone fusion with neighboring bones (*e.g.* ribs, radius and ulna)), along with joint and soft tissue swelling—which then resolves over the following months. To date all cases carry a single point mutation in *COL1A1* (c.3040C>T; p.Arg836Cys) (260). Mutations in *COL1A1* usually cause osteogenesis imperfecta; and the reason for the differing phenotype in Caffey’s disease is not known – nor is it known why this condition settles down over time. Interestingly, a *COL1A1* mutation has been identified in an Australian terrier with canine hyperostosis (261), and mutations in various solute carrier genes have been described in other cases of canine calvarial hyperostosis and craniomandibular osteopathies (which are likely overlapping conditions) (261). Whether mutations in these genes contribute to human diseases similarly is unknown.

Other forms of neonatal osteosclerosis include Blomstrand dysplasia (MIM215045), due to autosomal recessive inactivating mutations in *PTHR1* which codes for the PTH/PTHrP receptor 1, and which is usually lethal; desmosterolosis (MIM602398), due to autosomal recessive mutations in *DHCR24* which codes for 3‐beta‐hydroxysterol delta‐24‐reductase, with mutations resulting in impaired sterol‐metabolism; and Raine dysplasia (MIM259775), due to autosomal recessive *FAM20C* mutations coding for Dentin matrix protein 4, which can also be lethal.

## Polygenic Inheritance of High BMD

GWAS in populations selected due to their high BMD have identified novel BMD-determining loci relevant not only in the extreme population but also in the general population. In 2011, we performed the first extreme truncate selection GWAS of BMD (33), as the use of extreme cases and/or “super controls,” drawn from opposite ends of the same population distribution, maximizes statistical power (262). This was one of the first such extreme truncate selection GWAS for any phenotype; and such augmentation of statistical power through analysis of extreme phenotypes has since been shown to be advantageous in a range of clinical phenotypes (263–266) and is now an established approach to investigate the genetic architecture of complex disease (262, 267). In addition to replicating associations for 21 of the then 26 known BMD loci (identified from analyses of populations with normally distributed BMD) (268), we identified six new genetic associations in loci near *CLCN7, GALNT3, IBSP, LTBP3, RSPO3*, and *SOX4* (33), which subsequently replicated in larger general population GWAS (17, 18). This project highlighted the efficiency of extreme-truncated selection for quantitative trait GWAS design (33).

More recently, we conducted a GWAS of arguably the most extreme BMD population to date, identifying further two genome-wide significant SNPs, rs9292469 (48.5kb 3′ of *NPR3* with the LD block including part of this gene) and rs2697825 (within an intron of *SPON1*) associated with lumbar spine and hip BMD respectively. *NRP3* regulates endochondral ossification and skeletal growth (269–272), while *SPON1* modulates TGF-β-regulated BMP-driven osteoblast differentiation (273). *SPON1*, coding for an extracellular matrix glycoprotein, had not previously been associated with a bone phenotype in humans; however interestingly, *Spon1* knockout mice have a skeletal HBM phenotype (274). These novel loci are now under active investigation as future therapeutic targets.

## Translational Potential of Dissecting the Genetics of HBM

The working assumption underlying the efforts of ourselves and others in this field is that understanding the genetic architecture of skeletal diseases characterized by HBM will elucidate critical pathways involved in bone growth and regulation, and aid development of novel therapeutics to increase bone mass (275). Successful drug targets (*i.e.*, those for whom drugs have successfully passed through all development steps to an approved drug indication) are enriched with genes known to be involved in human disease, whether identified through common or rare variant analysis (276). Inspiringly for those of us who study bone, the concordance between disease indication and disease/pathway association (whether identified through rare or common variant studies) is strongest for drugs targeting the musculoskeletal system, compared with all other systems (including diabetes, autoimmunity, cardiovascular disease and oncology). Importantly, there is no relationship between genomic effect size and approved drug status, emphasizing the role of studying both rare variants of large effect and common variants of small effect (276).

The definitive proof-of-concept for this working hypothesis has been the development of antibodies to sclerostin, a protein only identified through analysis of HBM families with sclerosteosis and van Buchem’s disease (110–112), with completion of phase 3 clinical trials (147, 277) and the first-in-class agent (romosozumab) approved for clinical use by the US Food and Drugs Administration. Similarly, genetic dissection of pycnodysostosis led to the development of Cathepsin K inhibitors and the first-in-class agent (odanocatib) (207), successful in phase 3 and extension trials but disappointingly not taken forward into clinical practice (248). Although we acknowledge wholeheartedly that many medications currently used in osteoporosis, were not developed as a direct consequence of genetic studies, it is interesting to reflect that bisphosphonates, selective estrogen receptor modulators, estrogen, cathepsin K inhibitors, denosumab, anti-sclerostin antibodies and PTH and its analogs all target proteins associated with a monogenic bone condition; and, with the exception of bisphosphonates and cathepsin-K inhibitors [but with the potential addition of DKK-1 inhibitors, which have shown promise in murine models (278, 279)], all target genes in loci with common variant association with BMD. We await news of further Wnt pathway agonists, also in development, as novel anabolic treatments for osteoporosis (278–281).

## Concluding Comments: The Value of Studying Extreme Phenotypes

In the 17th century William Harvey acknowledged the potential benefits of studying the natural, but rarely occurring, extreme cases, in order that they might elucidate systems pertinent to the general population: “Nature is nowhere accustomed more openly to display her secret mysteries than in cases where she shows traces of her workings apart from the beaten path; nor is there any better way to advance the proper practice of medicine than to give our minds to the discovery of the usual law of Nature by careful investigation of cases of rare forms of disease” (282).

These words summarise the rationale that we (and others) have used in considering and investigating individuals with high BMD (15, 33). The genetic revolution—both sequencing and high-throughput microarray genotyping—has contributed greatly to the understanding of both common (17, 18) and rare (8) bone pathologies, with identification of multiple genes and critical pathways, leading already to the development of novel therapeutics. We would particularly like to highlight that progress in this field has been greatly enabled by collaboration and co-operation between centers and within consortia around the globe. However, as discussed above, the most common form of sclerosing dysplasia appears to be the currently unexplained HBM phenotype, with features suggestive of a mild skeletal dysplasia. Given past history in this field, it is highly likely that further genetic dissection of HBM cases will yield further novel insights into bone regulation; and it is our hope that this work will contribute to improved health for individuals with HBM and for other individuals with metabolic bone diseases.

## Author Contributions

All authors contributed to the article and approved the submitted version.

## Funding

CG received funding from Versus Arthritis (grant reference 20000).

## Conflict of Interest

The authors declare that the research was conducted in the absence of any commercial or financial relationships that could be construed as a potential conflict of interest.

## References

[1] Mendel JG Versucheüber Pflanzenhybriden”, Verhandlungen des naturforschenden Vereines in Brünn, Bd. IV für das Jahr, 1865, Abhandlungen: 3–47. For the English translation, see: Druery, C.T.; Bateson, William (1901). “Experiments in plant hybridization. J R Hortic Soc (1866) 26:1–32.

[2] Garrod AE The incidence of alkaptonuria: a study in chemical individuality. 1902. Mol Med (Cambridge Mass) (1996) 2(3):274–82. 10.1007/BF03401625 PMC2230159 8784780

[3] Fisher RA XV.—The Correlation between Relatives on the Supposition of Mendelian Inheritance. Trans R Soc Edinburgh (1919) 52(2):399–433. 10.1017/S0080456800012163

[4] Gratten J Wray NR Keller MC Visscher PM Large-scale genomics unveils the genetic architecture of psychiatric disorders. Nat Neurosci (2014) 17(6):782–90. 10.1038/nn.3708 PMC4112149 24866044

[5] Timpson NJ Greenwood CMT Soranzo N Lawson DJ Richards JB Genetic architecture: the shape of the genetic contribution to human traits and disease. Nat Rev Genet (2018) 19(2):110–24. 10.1038/nrg.2017.101 29225335

[6] Janssens AC van Duijn CM An epidemiological perspective on the future of direct-to-consumer personal genome testing. Invest Genet (2010) 1(1):10. 10.1186/2041-2223-1-10 PMC2990732 21092344

[7] World Health Organisation Human Genomics in Global Health: Genes and human diseases. World Health Organisation, Available at: https://www.who.int/genomics/public/geneticdiseases/en/index2.html.

[8] Mortier GR Cohn DH Cormier-Daire V Hall C Krakow D Mundlos S Nosology and classification of genetic skeletal disorders: 2019 revision. Am J Med Genet A (2019) 179(12):2393–419. 10.1002/ajmg.a.61366 31633310

[9] Visscher PM Hill WG Wray NR Heritability in the genomics era–concepts and misconceptions. Nat Rev Genet (2008) 9(4):255–66. 10.1038/nrg2322 18319743

[10] Visscher PM Goddard ME Derks EM Wray NR Evidence-based psychiatric genetics, AKA the false dichotomy between common and rare variant hypotheses. Mol Psychiatry (2012) 17(5):474–85. 10.1038/mp.2011.65 21670730

[11] Visscher PM Brown MA McCarthy MI YmcCarthy MI Yang J Five Years of GWAS Discovery. Am J Hum Gen (2012) 90(1):7–24. 10.1016/j.ajhg.2011.11.029 PMC3257326 22243964

[12] Kuchenbaecker KB McGuffog L Barrowdale D Lee A Soucy P Joe D Evaluation of Polygenic Risk Scores for Breast and Ovarian CancerRisk Prediction in BRCA1 and BRCA2 Mutation Carriers. J Natl CancerInst (2017) 109(7):1–15. 10.1093/jnci/djw302 PMC5408990 28376175

[13] Brown MA Li Z Cao KL Biomarker development for axial spondyloarthritis. Nat Rev Rheumatol (2020) 16(8):448–63. 10.1038/s41584-020-0450-0 32606474

[14] Li Z Genetic risk score prediction in ankylosing spondylitis [abstract]. Arthritis Rheumatol (2018) 70:836.

[15] Gregson CL Newell F Leo PJ Clark GR Paternoster L Marshall M Genome-wide association study of extreme high bone mass: Contribution of common genetic variation to extreme BMD phenotypes and potential novel BMD-associated genes. Bone (2018) 114:62–71. 10.1016/j.bone.2018.06.001 29883787 PMC6086337

[16] Estrada K Styrkarsdottir U Evangelou E Yi Hsiang H Duncan EL Ntzani EE Genome-wide meta-analysis identifies 56 bone mineral density lociand reveals 14 loci associated with risk of fracture. Nat Genet(2012)44(5):491–501. 10.1038/ng.2249 22504420 PMC3338864

[17] Morris JA Kemp JP Youlten SE Laurent L Logan JG Chai RC An atlas of genetic influences on osteoporosis in humans andmice. Nat Genet (2019)51:258–66. 10.1038/s41588-018-0302-x PMC6358485 30598549

[18] Kemp JP Morris JA Medina-Gomez C Forgetta V Warrington NM Youlten SE Identification of 153 new loci associated with heel bone mineraldensity and functional involvement of GPC6 in osteoporosis. NatGenet (2017) 49(10):1468–75 10.1038/ng.3949 PMC5621629 28869591

[19] Lambert SA Abraham G Inouye M Towards clinical utility of polygenic risk scores. Hum Mol Genet (2019) 28(R2):R133–r42. 10.1093/hmg/ddz187 31363735

[20] Schrodi SJ Mukherjee S Shan Y Tromp G Sninsky JJ Callear AP Genetic-based prediction of disease traits: prediction is very difficult, especially about the future. Front Genet (2014) 5:162. 10.3389/fgene.2014.00162 24917882 PMC4040440

[21] Wray NR Yang J Goddard ME Visscher PM The genetic interpretation of area under the ROC curve in genomic profiling. PLoS Genet (2010) 6(2):e1000864. 10.1371/journal.pgen.1000864 20195508 PMC2829056

[22] Leo PJ Madeleine MM Wang S Schwartz SM Newell F Pettersson-Kymmer U Defining the genetic susceptibility to cervical neoplasia-A genome-wide association study. PLoS Genet (2017) 13(8):e1006866. 10.1371/journal.pgen.1006866 28806749 PMC5570502

[23] Li R Chen Y Ritchie MD Moore JH Electronic health records and polygenic risk scores for predicting disease risk. Nat Rev Genet (2020) 21(8):493–502. 10.1038/s41576-020-0224-1 32235907

[24] Khera AV Chaffin M Aragam KG Haas ME Roselli C Choi SH Genome-wide polygenic scores for common diseases identify individuals with risk equivalent to monogenic mutations. Nat Genet (2018) 50(9):1219–24. 10.1038/s41588-018-0183-z PMC6128408 30104762

[25] Johnell O Kanis JA Oden A Johansson H De, Laet C Delmas P Predictive value of BMD for hip and other fractures. J Bone Miner Res (2005) 20(7):1185–94. 10.1359/JBMR.050304 15940371

[26] World Health Organization Assessment of fracture risk and its application to screening for postmenopausal osteoporosis: report of a WHO study group. (1994) 843:1–129. Available at: https://apps.who.int/iris/handle/10665/39142 7941614

[27] Kanis JA Johnell O De Laet C Johansson H Oden A Delmas P A meta-analysis of previous fracture and subsequent fracture risk. Bone (2004) 35:375–82. 10.1016/j.bone.2004.03.024 15268886

[28] Pasco J Seeman E Henry M Merriman E Nicholson G Kotowicz M The population burden of fractures originates in women with osteopenia, not osteoporosis. Osteo Int (2006) 17(9):1404–9. 10.1007/s00198-006-0135-9 16699736

[29] Kanis JA Johnell O Oden A Johansson H McCloskey E FRAX and the assessment of fracture probability in men and women from the UK. Osteo Int (2008) 19(4):385–97. 10.1007/s00198-007-0543-5 PMC2267485 18292978

[30] Whyte MP Misinterpretation of osteodensitometry with high bone density: BMD Z > or = + 2.5 is not “normal”. J Clin Densitom (2005) 8(1):1–6. 10.1385/JCD:8:1:001 15722580

[31] Bassett JHD Gogakos A White JK Evans H Jacques RM van der Spek AH Rapid-Throughput Skeletal Phenotyping of 100 Knockout Mice Identifies 9 New Genes That Determine Bone Strength. PLoS Genet (2012) 8(8):e1002858. 10.1371/journal.pgen.1002858 22876197 PMC3410859

[32] Morin S Leslie W High bone mineral density is associated with high body mass index. Osteo Int (2009) 20(7):1267–71. 10.1007/s00198-008-0797-6 19034375

[33] Duncan EL Danoy P Kemp JP Leo PJ McCloskey E Nicholson GC Genome-Wide Association Study Using Extreme Truncate Selection Identifies Novel Genes Affecting Bone Mineral Density and Fracture Risk. PLoS Genet (2011) 7(4):e1001372. 10.1371/journal.pgen.1001372 21533022 PMC3080863

[34] Gregson CL Steel SA O’Rourke KP Allan K Ayuk J Bhalla A ‘Sink or swim’: an evaluation of the clinical characteristics of individuals with high bone mass. Osteo Int (2012) 23(2):643–54. 10.1007/s00198-011-1603-4 PMC3261396 21455762

[35] White J Yeats A Skipworth G Tables for Statisticians. Cheltenham: Stanley Thornes (1979).

[36] Gregson CL Hardcastle SA Cooper C Tobias JH Friend or foe: high bone mineral density on routine bone density scanning, a review of causes and management. Rheumatology (2013) 52(6):968–85. 10.1093/rheumatology/ket007 PMC3651616 23445662

[37] Zengini E Hatzikotoulas K Tachmazidou I Steinberg J Hartwig FP Southam L Genome-wide analyses using UK Biobank data provide insights into the genetic architecture of osteoarthritis. Nat Genet (2018) 50(4):549–58. 10.1038/s41588-018-0079-y PMC5896734 29559693

[38] Westerveld LA Verlaan JJ Lam MGEH Scholten WP Bleys RLAW Dhert WJA The influence of diffuse idiopathic skeletal hyperostosis on bone mineral density measurements of the spine. Rheumatology (2009) 48(9):1133–6. 10.1093/rheumatology/kep177 19605371

[39] Couto AR Brown MA Genetic factors in the pathogenesis of CPPD crystal deposition disease. Curr Rheumatol Rep (2007) 9(3):231–6. 10.1007/s11926-007-0037-7 17531177

[40] Eser P Bonel H Seitz M Villiger PM Aeberli D Patients with diffuse idiopathic skeletal hyperostosis do not have increased peripheral bone mineral density and geometry. Rheumatology (2010) 49(5):977–81. 10.1093/rheumatology/keq014 20156975

[41] Weinfeld RM Olson PN Maki DD Griffiths HJ The prevalence of diffuse idiopathic skeletal hyperostosis (DISH) in two large American Midwest metropolitan hospital populations. Skeletal Radiol (1997) 26(4):222–5. 10.1007/s002560050225 9151370

[42] Ellinghaus D Jostins L Spain SL Cortes A Bethune SL Han B Analysis of five chronic inflammatory diseases identifies 27 newassociations and highlights disease-specific patterns at shared loci. NatGenet (2016)48(5):510–8. 10.1038/ng.3528 PMC4848113 26974007

[43] Muntean L Rojas-Vargas M Font P Simon SP Rednic S Schiotis R Relative value of the lumbar spine and hip bone mineral density and bone turnover markers in men with ankylosing spondylitis. Clin Rheumatol (2011) 30(5):691–5. 10.1007/s10067-010-1648-3 21221691

[44] Natarajan P Bis JC Bielak LF Cox AJ Dörr LF Feitosa MF Multiethnic Exome-Wide Association Study of SubclinicalAtherosclerosis. Circ Cardiovasc Genet (2016)9(6):511–20 10.1161/CIRCGENETICS.116.001572 PMC5418659 27872105

[45] Bergen AA Plomp AS Schuurman EJ Terry S Breuning M Dauwerse H Mutations in ABCC6 cause pseudoxanthoma elasticum. Nat Genet (2000) 25(2):228–31. 10.1038/76109 10835643

[46] Le Saux O Urban Z Tschuch C Csiszar K Bacchelli B Quaglino D Mutations in a gene encoding an ABC transporter cause pseudoxanthoma elasticum. Nat Genet (2000) 25(2):223–7. 10.1038/76102 10835642

[47] Ringpfeil F Pulkkinen L Uitto J Molecular genetics of pseudoxanthoma elasticum. Exp Dermatol (2001) 10(4):221–8. 10.1034/j.1600-0625.2001.100401.x 11493310

[48] Rutsch F Nitschke Y Terkeltaub R Genetics in arterial calcification: pieces of a puzzle and cogs in a wheel. Circ Res (2011) 109(5):578–92. 10.1161/CIRCRESAHA.111.247965 PMC3248761 21852556

[49] Yildiz M Canatan D Soft tissue density variations in thalassemia major: a possible pitfall in lumbar bone mineral density measurements by dual-energy X-ray absorptiometry. Pediatr HematolOncol (2005) 22(8):723–6. 10.1080/08880010500278707 16251180

[50] Cao A Galanello R Beta-thalassemia. Genet Med (2010) 12(2):61–76. 10.1097/GIM.0b013e3181cd68ed 20098328

[51] Johnson PH Weinreb NJ Cloyd JC Tuite PJ Kartha RV GBA1 mutations: Prospects for exosomal biomarkers in α-synuclein pathologies. Mol Genet Metab (2020) 129(2):35–46. 10.1016/j.ymgme.2019.10.006 31761523 PMC7002237

[52] Spencer RP Szigeti DP Abdominal abscess detected by lumbar bone densitometry examination. Clin NuclMed (1998) 23(1):44. 10.1097/00003072-199801000-00016 9442969

[53] Smith JA Spencer RP Szigeti DP Gall stones detected on lumbar bone densitometry examination. J Clin Densitom (1998) 1(4):403–4. 10.1385/JCD:1:4:403 15304888

[54] Bazzocchi A Ferrari F Diano D Albisinni U Battista G Rossi C Incidental Findings with Dual-Energy X-Ray Absorptiometry: Spectrum of Possible Diagnoses. Calcif Tiss Int (2012) 91(2):149–56. 10.1007/s00223-012-9609-2 22623178

[55] Howles SA Thakker RV Genetics of kidney stone disease. Nat Rev Urol (2020) 17(7):407–21. 10.1038/s41585-020-0332-x 32533118

[56] Hauache OM Vieira JG Alonso G Martins LR Brandao C Increased hip bone mineral density in a woman with gluteal silicon implant. J Clin Densitom (2000) 3(4):391–3. 10.1385/JCD:3:4:391 11175920

[57] Spencer RP Szigeti DP Engin IO Effect of laminectomy on measured bone density. J Clin Densitom (1998) 1(4):375–7. 10.1385/JCD:1:4:375 15304884

[58] Pomerantz MM Freedman ML The genetics of cancer risk. Cancer J (2011) 17(6):416–22. 10.1097/PPO.0b013e31823e5387 PMC3934208 22157285

[59] Takigawa T Tanaka M Nakanishi K Misawa H Sugimoto Y Takahata T SAPHO syndrome associated spondylitis. Eur Spine J (2008) 17(10):1391–7. 10.1007/s00586-008-0722-x PMC2556478 18642032

[60] Laredo JD Vuillemin-Bodaghi V Boutry N Cotten A Parlier-Cuau C SAPHO syndrome: MR appearance of vertebral involvement. Radiology (2007) 242(3):825–31. 10.1148/radiol.2423051222 17244716

[61] Liu S Tang M Cao Y Li C Synovitis, acne, pustulosis, hyperostosis, and osteitis syndrome: review and update. Ther Adv Musculoskelet Dis (2020) 12:1759720X20912865–1759720X. 10.1177/1759720X20912865 PMC7236399 32523634

[62] El-Feky M Gaillard F Rugger jersey spine (hyperparathyroidism). Radiopaedia. (2020) Available at: http://radiopaedia.org/articles/rugger-jersey-spine-hyperparathyroidism-1?lang=gb

[63] Rasuli B Gaillard F Renal osteodystrophy. Radiopaedia. (2020) Available at: https://radiopaedia.org/articles/renal-osteodystrophy?lang=gb

[64] Ketteler M Leonard MB Block GA Shroff R Evenepoel P Tonelli MA KDIGO 2017 Clinical Practice Guideline Update for the Diagnosis, Evaluation, Prevention, and Treatment of Chronic Kidney Disease-Mineral and Bone Disorder (CKD-MBD). Kidney Int Suppl (2011) (2017) 7(1):1–59. 10.1016/j.kisu.2017.04.001 30675420 PMC6340919

[65] Gennari L Rendina D Falchetti A Merlotti D Paget’s Disease of Bone. Calcif Tissue Int (2019) 104(5):483–500. 10.1007/s00223-019-00522-3 30671590

[66] Albagha OME Wani SE Visconti MR Alonso N Goodman K Brandi ML Genome-wide association identifies three new susceptibility loci for Paget’s disease of bone. Nat Genet (2011) 43(7):685–9. 10.1038/ng.845 21623375

[67] Ralston SH Taylor JP Rare Inherited forms of Paget’s Disease and Related Syndromes. Calcif Tissue Int (2019) 104(5):501–16. 10.1007/s00223-019-00520-5 PMC6779132 30756140

[68] Reid IR Murphy WA Hardy DC Teitelbaum SL Bergfeld MA Whyte MP X-linked hypophosphatemia: skeletal mass in adults assessed by histomorphometry, computed tomography, and absorptiometry. Am J Med (1991) 90(1):63–9. 10.1016/0002-9343(91)90507-T 1986592

[69] Cundy T Dray M Delahunt J Hald JD Langdahl B Li C Mutations That Alter the Carboxy-Terminal-Propeptide Cleavage Site of the Chains of Type I Procollagen Are Associated With a Unique Osteogenesis Imperfecta Phenotype. J Bone Miner Res (2018) 33(7):1260–71. 10.1002/jbmr.3424 PMC6031457 29669177

[70] McInerney-Leo AM Duncan EL Leo PJ Gardiner B Bradbury LA Harris JE COL1A1 C-propeptide cleavage site mutation causes high bone mass, bone fragility and jaw lesions: a new cause of gnathodiaphyseal dysplasia? Clin Genet (2015) 88(1):49–55. 10.1111/cge.12440 24891183

[71] Tsutsumi S Kamata N Vokes TJ Maruoka Y Nakakuki K Enomoto S The novel gene encoding a putative transmembrane protein is mutated in gnathodiaphyseal dysplasia (GDD). Am J Hum Genet (2004) 74(6):1255–61. 10.1086/421527 PMC1182089 15124103

[72] Wang Y Yin Y Gilula LA Wilson AJ Endemic fluorosis of the skeleton: radiographic features in 127 patients. Am J Roentgenol (1994) 162(1):93–8. 10.2214/ajr.162.1.8273699 8273699

[73] Hallanger Johnson JE Kearns AE Doran PM Khoo TK Wermers RA Fluoride-related bone disease associated with habitual tea consumption. Mayo Clin Proc (2007) 82(6):719–24. 10.4065/82.6.719 17550752

[74] Joshi S Hlaing T Whitford GM Compston JE Skeletal fluorosis due to excessive tea and toothpaste consumption. Osteo Int (2010) 22:2557–60. 10.1007/s00198-010-1428-6 20936399

[75] Kaji H Sugimoto T Nakaoka D Okimura Y Kaji H Abe H Bone metabolism and body composition in Japanese patients with active acromegaly. Clin Endocrinol (Oxf) (2001) 55(2):175–81. 10.1046/j.1365-2265.2001.01280.x 11531923

[76] Gadelha MR Kasuki L Korbonits M The genetic background of acromegaly. Pituitary (2017) 20(1):10–21. 10.1007/s11102-017-0789-7 28161730 PMC5334425

[77] Fiore CE Riccobene S Mangiafico R Santoro F Pennisi P Hepatitis C-associated osteosclerosis (HCAO): report of a new case with involvement of the OPG/RANKL system. Osteoporos Int (2005) 16(12):2180–4. 10.1007/s00198-005-1858-8 15983730

[78] Manganelli P Giuliani N Fietta P Mancini C Lazzaretti M Pollini A OPG/RANKL system imbalance in a case of hepatitis C-associated osteosclerosis: the pathogenetic key? Clin Rheumatol (2005) 24(3):296–300. 10.1007/s10067-004-1031-3 15583970

[79] Schwartz KM Skinner JA Hepatitis C-associated osteosclerosis: a case report. Skeletal Radiol (2008) 37(7):679–81. 10.1007/s00256-008-0471-2 18414851

[80] Rollison DE Howlader N Smith MT Strom SS Merritt WD Ries LA Epidemiology of myelodysplastic syndromes and chronic myeloproliferative disorders in the United States, 2001-2004, using data from the NAACCR and SEER programs. Blood (2008) 112(1):45–52. 10.1182/blood-2008-01-134858 18443215

[81] Diamond T Smith A Schnier R Manoharan A Syndrome of myelofibrosis and osteosclerosis: a series of case reports and review of the literature. Bone (2002) 30(3):498–501. 10.1016/S8756-3282(01)00695-0 11882464

[82] Gangat N Tefferi A Myelofibrosis biology and contemporary management. Br J Haematol (2020) 191:152–70. 10.1111/bjh.16576 32196650

[83] Barete S Assous N de Gennes C Grandpeix C Feger F Palmerini F Systemic mastocytosis and bone involvement in a cohort of 75patients. Ann Rheum Dis (2010) 69(10):1838–41. 10.1136/ard.2009.124511 20570833

[84] Kushnir-Sukhov NM Brittain E Reynolds JC Akin C Metcalfe DD Elevated tryptase levels are associated with greater bone density in a cohort of patients with mastocytosis. Int Arch Allergy Immunol (2006) 139(3):265–70. 10.1159/000091172 16449817

[85] Martelli M Monaldi C De Santis S Bruno S Mancini M Soverini S Recent Advances in the Molecular Biology of Systemic Mastocytosis:Implications for Diagnosis, Prognosis, and Therapy. Int J Mol Sci (2020) 21(11) 21(11): 3987. 10.3390/ijms21113987 PMC7312790 32498255

[86] Vedi S Purdie DW Ballard P Bord S Cooper AC Compston JE Bone remodeling and structure in postmenopausal women treated with long-term, high-dose estrogen therapy. Osteoporos Int (1999) 10(1):52–8. 10.1007/s001980050194 10501780

[87] Styrkarsdottir U Helgason H Sigurdsson A Norddahl GL Agustsdottir AB Reynard LN Whole-genome sequencing identifies rare genotypes in COMP and CHADL associated with high risk of hip osteoarthritis. Nat Genet (2017) 49(5):801–5. 10.1038/ng.3816 28319091

[88] Brown MA Kennedy LG MacGregor AJ Darke C Duncan E Shatford JL Susceptibility to ankylosing spondylitis in twins: the role of genes, HLA, and the environment. Arthritis Rheum (1997) 40(10):1823–8. 10.1002/art.1780401015 9336417

[89] Ognjenovic M Raymond W Inderjeeth C Keen H Preen D Nossent J The risk and consequences of vertebral fracture in patients with Ankylosing Spondylitis: a population-based data linkage study. J Rheumatol (2020). 10.3899/jrheum.190675 32062601

[90] Corkill M Topless R Worthington A Mitchell R Gregory K Stamp LK Exploring the Relationship between Gout and Diffuse Idiopathic Skeletal Hyperostosis (DISH): An Epidemiologic and Genetic Study [abstract]. Arthritis Rheumatol (2018) 70(2225).

[91] Terayama K Genetic studies on ossification of the posterior longitudinal ligament of the spine. Spine (1989) 14(11):1184–91. 10.1097/00007632-198911000-00009 2513651

[92] Nakajima M Takahashi A Tsuji T A genome-wide association study identifies susceptibility loci for ossification of the posterior longitudinal ligament of the spine. Nat Genet (2014) 46(9):1012–6. 10.1038/ng.3045 25064007

[93] Chen X Guo J Cai T Zhang F Pan S Zhang L Targeted next-generation sequencing reveals multiple deleterious variants in OPLL-associated genes. Sci Rep (2016) 6:26962. 10.1038/srep26962 27246988 PMC4887887

[94] Drinka PJ DeSmet AA Bauwens SF Rogot A The effect of overlying calcification on lumbar bone densitometry. Calcif Tissue Int (1992) 50(6):507–10. 10.1007/BF00582163 1525705

[95] Masud T Langley S Wiltshire P Doyle DV Spector TD Effect of spinal osteophytosis on bone mineral density measurements in vertebral osteoporosis. BMJ (1993) 307(6897):172–3. 10.1136/bmj.307.6897.172 PMC1678366 8343746

[96] Orwoll ES Oviatt SK Mann T The impact of osteophytic and vascular calcifications on vertebral mineral density measurements in men. J Clin Endo Metab (1990) 70(4):1202–7. 10.1210/jcem-70-4-1202 2318940

[97] Rand T Seidl G Kainberger F Resch A Hittmair K Schneider B Impact of spinal degenerative changes on the evaluation of bone mineral density with dual energy X-ray absorptiometry (DXA). Calcif Tissue Int (1997) 60(5):430–3. 10.1007/s002239900258 9115160

[98] Bucay N Sarosi I Dunstan CR Morony S Tarpley J Capparelli C osteoprotegerin-deficient mice develop early onset osteoporosis and arterial calcification. Genes Dev (1998) 12(9):1260–8. 10.1101/gad.12.9.1260 PMC316769 9573043

[99] Lewis JR Eggermont CJ Schousboe JT Lim WH Wong G Khoo B Association Between Abdominal Aortic Calcification, Bone Mineral Density, and Fracture in Older Women. J Bone Miner Res (2019) 34(11):2052–60. 10.1002/jbmr.3830 31310354

[100] Cooper C Harvey NC Dennison EM van Staa TP Update on the epidemiology of Paget’s disease of bone. J Bone Miner Res (2006) 21:3–8. 10.1359/jbmr.06s201 17229005

[101] Dell’Atti C Cassar-Pullicino VN Lalam RK Tins BJ Tyrrell PN The spine in Paget’s disease. Skeletal Radiol (2007) 36(7):609–26. 10.1007/s00256-006-0270-6 PMC1934928 17410356

[102] Rowland Hogue CJ Getting to the Why. Epidemiology (1997) 8(3):230. 9115014

[103] Whyte MP Paget’s Disease of Bone and Genetic Disorders of RANKL/OPG/RANK/NF-κB Signaling. Ann N Y Acad Sci (2006) 1068(1):143–64. 10.1196/annals.1346.016 16831914

[104] Kim JH Kim K Kim I Seong S Kim SW Kim N Role of anoctamin 5, a gene associated with gnathodiaphyseal dysplasia, in osteoblast and osteoclast differentiation. Bone (2019) 120:432–8. 10.1016/j.bone.2018.12.010 30557634

[105] Riggs BL Hodgson SF O’Fallon WM Chao EY Wahner HW Muhs JM Effect of fluoride treatment on the fracture rate in postmenopausal women with osteoporosis. N Eng J Medi (1990) 322(12):802–9. 10.1056/NEJM199003223221203 2407957

[106] Kleerekoper M Peterson EL Nelson DA Phillips E Schork MA Tilley BC A randomized trial of sodium fluoride as a treatment for postmenopausal osteoporosis. Osteoporos Int (1991) 1(3):155–61. 10.1007/BF01625446 1790403

[107] De Ridder R Boudin E Mortier G Van Hul W Human Genetics of Sclerosing Bone Disorders. Curr Osteoporos Rep (2018) 16(3):256–68. 10.1007/s11914-018-0439-7 29656376

[108] Baron R Kneissel M WNT signaling in bone homeostasis and disease: from human mutations to treatments. Nat Med (2013) 19(2):179–92. 10.1038/nm.3074 23389618

[109] Fijalkowski I Geets E Steenackers E Van Hoof V Ramos FJ Mortier G A Novel Domain-Specific Mutation in a Sclerosteosis Patient Suggests a Role of LRP4 as an Anchor for Sclerostin in Human Bone. J Bone mineral Res Off J Am Soc Bone Mineral Res (2016) 31(4):874–81. 10.1002/jbmr.2782 26751728

[110] Staehling-Hampton K Proll S Paeper BW Zhao L Charmley P Brown A A 52-kb deletion in the SOST-MEOX1 intergenic region on 17q12-q21 is associated with van Buchem disease in the Dutch population. Am J Med Genet (2002) 110(2):144–52. 10.1002/ajmg.10401 12116252

[111] Balemans W Patel N Ebeling M Van Hul E Wuyts W Lacza C Identification of a 52 kb deletion downstream of the SOST gene in patients with van Buchem disease. J Med Genet (2002) 39(2):91–7. 10.1136/jmg.39.2.91 PMC1735035 11836356

[112] van Oers RF Van RB Ito K Hilbers PA Huiskes R. A sclerostin-based theory for strain-induced bone formation. Biomech Model Mechanobiol (2011) 10(5):663–70. 10.1007/s10237-010-0264-0 21069416

[113] Hamersma H Gardner J Beighton P The natural history of sclerosteosis. Clin Genet (2003) 63(3):192–7. 10.1034/j.1399-0004.2003.00036.x 12694228

[114] Hansen HG Opitz H Schmid F Handbuch der Kinderheilkunde. Berlin: Springer (2010).

[115] Brunkow ME Gardner JC Van Ness J Paeper BW Kovacevich BR Proll S Bone Dysplasia Sclerosteosis Results from Loss of the SOST Gene Product, a Novel Cystine Knot-Containing Protein. Am J Med Genet (2001) 68(3):577–89. 10.1086/318811 PMC1274471 11179006

[116] Van Buchem FS Hadders HN Ubbens R An uncommon familial systemic disease of the skeleton: hyperostosis corticalis generalisata familiaris. Acta Radiol (1955) 44(2):109–20. 10.1177/028418515504400203 13258333

[117] Fosmoe RJ Holm RS Hildreth RC Van Buchem’s disease (hyperostosis corticalis generalisata familiaris). A case report. Radiology (1968) 90(4):771–4. 10.1148/90.4.771 4867898

[118] Whyte MP Deepak Amalnath S McAlister WH Pedapati R Muthupillai V Duan S Sclerosteosis: Report of type 1 or 2 in three Indian Tamil families and literature review. Bone (2018) 116:321–32. 10.1016/j.bone.2018.07.022 30077757

[119] Leupin O Piters E Halleux C Hu S Kramer I Morvan F Bone Overgrowth-associated Mutations in the LRP4 Gene Impair Sclerostin Facilitator Function. J Biolog Chem (2011) 286(22):19489–500. 10.1074/jbc.M110.190330 PMC3103328 21471202

[120] Johnson ML Gong G Kimberling W Recker SM Kimmel DB Recker RB Linkage of a gene causing high bone mass to human chromosome 11 (11q12-13). Am J Hum Genet (1997) 60(6):1326–32. 10.1086/515470 PMC1716125 9199553

[121] Little RD Carulli JP Del Mastro RG Dupuis J Osborne M Folz C A mutation in the LDL receptor-related protein 5 gene results in the autosomal dominant high-bone-mass trait. Am J Hum Genet (2002) 70(1):11–9. 10.1086/338450 PMC419982 11741193

[122] Koay A Brown MA Genetic disorders of the LRP5-Wnt signalling pathway affecting the skeleton. Trends Mol Med (2005) 11:129–37. 10.1016/j.molmed.2005.01.004 15760771

[123] Rickels MR Zhang X Mumm S Whyte MP Oropharyngeal skeletal disease accompanying high bone mass and novel LRP5 mutation. J Bone Miner Res (2005) 20(5):878–85. 10.1359/JBMR.041223 15824861

[124] Boyden LM Mao J Belsky J Mitzner L Farhi A Mitnick MA High bone density due to a mutation in LDL-receptor-related protein 5. N Eng J Med (2002) 346(20):1513–21. 10.1056/NEJMoa013444 12015390

[125] Whyte MP Reinus WH Mumm S High-bone mass disease and LRP5. N Eng J Med (2004) 350:2096–9. 10.1056/NEJM200405133502017 15141052

[126] Boyden L Insogna K Lifton R High Bone Mass Disease and LRP5. N Eng J Med (2004) 350(20):2098–9. 10.1056/NEJM200405133502017 15141052

[127] Van Wesenbeeck L Cleiren E Gram J Beals RK Benichou O Scopelliti D Six novel missense mutations in the LDL receptor-related protein 5 (LRP5) gene in different conditions with an increased bone density. Am J Hum Genet (2003) 72(3):763–71. 10.1086/368277 PMC1180253 12579474

[128] VanHul E Gram J Bollerslev J VanWesenbeeck L Mathysen D Andersen PE Localization of the gene causing autosomal dominant osteopetrosis type I to chromosome 11q12-13. J Bone Miner Res (2002) 17(6):1111–7. 10.1359/jbmr.2002.17.6.1111 12054167

[129] Kwee ML Balemans W Cleiren E Gille JJ Van Der Blij F Sepers JM An autosomal dominant high bone mass phenotype in association with craniosynostosis in an extended family is caused by an LRP5 missense mutation. J Bone Miner Res (2005) 20(7):1254–60. 10.1359/JBMR.050303 15940380

[130] Renton T Odell E Drage NA Differential diagnosis and treatment of autosomal dominant osteosclerosis of the mandible. Br J Oral Maxillofac Surg (2002) 40(1):55–9. 10.1054/bjom.2001.0719 11883972

[131] Bollerslev J Nielsen HK Larsen HF Mosekilde L Biochemical evidence of disturbed bone metabolism and calcium homeostasis in two types of autosomal dominant osteopetrosis. Acta Med Scand (1988) 224(5):479–83. 10.1111/j.0954-6820.1988.tb19614.x 3264447

[132] Bollerslev J Andersen PE Jr Radiological, biochemical and hereditary evidence of two types of autosomal dominant osteopetrosis. Bone (1988) 9(1):7–13. 10.1016/8756-3282(88)90021-X 3377922

[133] Beals RK Endosteal hyperostosis. J Bone Joint Surg Am (1976) 58(8):1172–3. 10.2106/00004623-197658080-00028 1002767

[134] Beals RK McLoughlin SW Teed RL McDonald C Dominant endosteal hyperostosis. Skeletal characteristics and review of the literature. J Bone Joint Surg Am (2001) 83-A(11):1643–9. 10.2106/00004623-200111000-00004 11701785

[135] Scopelliti D Orsini R Ventucci E Carratelli D [Van Buchem disease. Maxillofacial changes, diagnostic classification and general principles of treatment]. Minerva Stomatol (1999) 48(5):227–34. 10434540

[136] vanWesenbeeck L Odgren PR Mackay CA Van Hul W Localization of the gene causing the osteopetrotic phenotype in the incisors absent (ia) rat on chromosome 10q32.1. J Bone Miner Res (2004) 19(2):183–9. 10.1359/jbmr.2004.19.2.183 14969387

[137] Balemans W Devogelaer JP Cleiren E Piters E Caussin E Van Hul W Novel LRP5 missense mutation in a patient with a high bone mass phenotype results in decreased DKK1-mediated inhibition of Wnt signaling. J Bone Miner Res (2007) 22(5):708–16. 10.1359/jbmr.070211 17295608

[138] Whyte MP McAlister WH Zhang F Bijanki VN Nenninger A Gottesman GS New explanation for autosomal dominant high bone mass: Mutation of low-density lipoprotein receptor-related protein 6. Bone (2019) 127:228–43. 10.1016/j.bone.2019.05.003 31085352

[139] Whyte MP Gottesman GS Lin EL Mcalister WH Nenninger A Bijanki VN LRP6 Mutation: A New Cause of Autosomal Dominant High Bone Mass. (LB-1172) American Society for Bone and Mineral Research (ASBMR) 2018 Annual Meeting; 2018. Montreal, Canada (2018).

[140] Gregson CL Bergen DJM Leo P Sessions RB Wheeler L Hartley A A Rare Mutation in SMAD9 Associated With High Bone Mass Identifies the SMAD-Dependent BMP Signaling Pathway as a Potential Anabolic Target for Osteoporosis. J Bone Miner Res (2020) 35(1):92–105. 10.1002/jbmr.3875 31525280 PMC7004081

[141] Nurnberg P Tinschert S Mrug M Hampe J Muller CR Fuhrmann E The Gene for Autosomal Dominant Craniometaphyseal Dysplasia Maps to Chromosome 5p and Is Distinct from the Growth Hormone-Receptor Gene. Am J Hum Genet (1997) 61(4):918–23. 10.1086/514880 PMC1716005 9382103

[142] Reichenberger E Tiziani V Watanabe S Park L Ueki Y Santanna C Autosomal Dominant Craniometaphyseal Dysplasia Is Caused by Mutations in the Transmembrane Protein ANK. Am J Hum Genet (2001) 68(6):1321–6. 10.1086/320612 PMC1226118 11326338

[143] González-Rodríguez JD Luis-Yanes MI Inglés-Torres E Arango-Sancho P Cabrera-Sevilla JE Duque-Fernández MR Can acetazolamide be used to treat diseases involving increased bone mineral density? Intractable Rare Dis Res (2016) 5(4):284–9. 10.5582/irdr.2016.01067 PMC5116865 27904825

[144] Majewski F Lenz–Majewski hyperostotic dwarfism: Reexamination of the original patient. Am J Med Genet (2000) 93(4):335–8. 10.1002/1096-8628(20000814)93:4<335::AID-AJMG14>3.0.CO;2-5 10946362

[145] Piard J Lespinasse J Vlckova M Mensah MA Iurian S Simandlova M Cutis laxa and excessive bone growth due to de novo mutations in PTDSS1. Am J Med Genet A (2018) 176(3):668–75. 10.1002/ajmg.a.38604 PMC5838527 29341480

[146] Garcia-Garcia AS Martinez-Gonzalez JM Gomez-Font R Soto-Rivadeneira A Oviedo-Roldan L Current status of the torus palatinus and torus mandibularis. Med Oral Patol Oral Cir Bucal (2010) 15(2):e353–e60. 10.4317/medoral.15.e353 19767716

[147] McClung MR Grauer A Boonen S Bolognese MA Brown JP Diez-Perez A Romosozumab in Postmenopausal Women with Low Bone Mineral Density. N Eng J Med (2014) 370(5):412–20. 10.1056/NEJMoa1305224 24382002

[148] McClung MR Brown JP Diez-Perez A Resch H Caminis J Meisner P Effects of 24 Months of Treatment With Romosozumab Followed by 12 Months of Denosumab or Placebo in Postmenopausal Women With Low Bone Mineral Density: A Randomized, Double-Blind, Phase 2, Parallel Group Study. J Bone Miner Res (2018) 33(8):1397–406. 10.1002/jbmr.3452 29694685

[149] Stephen LX Hamersma H Gardner J Beighton P Dental and oral manifestations of sclerosteosis. Int DentJ (2001) 51(4):287–90. 10.1002/j.1875-595X.2001.tb00840.x 11570544

[150] Balemans W Van Den Ende J Freire Paes-Alves A Dikkers FG Willems PJ Vanhoenacker F Localization of the Gene for Sclerosteosis to the van Buchem Disease-Gene Region on Chromosome 17q12-q21. Am J Hum Genet (1999) 64(6):1661–9. 10.1086/302416 PMC1377909 10330353

[151] Tacconi P Ferrigno P Cocco L Carinas A Tamburini G Bergonzi P Sclerosteosis: report of a case in a black African man. Clin Genet (1998) 53(6):497–501. 10.1111/j.1399-0004.1998.tb02603.x 9712543

[152] Van Hul W Balemans W Van Hul E Dikkers FG Obee H Stokroos RJ Van Buchem disease (hyperostosis corticalis generalisata) maps to chromosome 17q12-q21. Am J Hum Genet (1998) 62(2):391–9. 10.1086/301721 PMC1376897 9463328

[153] van Lierop AHJM Hamdy NAT Papapoulos SE Glucocorticoids are not always deleterious for bone. J Bone Min Res (2010) 25(12):2520–4. 10.1002/jbmr.151 20549703

[154] Gong Y Vikkula M Boon L Liu J Beighton P Ramesar R Osteoporosis-pseudoglioma syndrome, a disorder affecting skeletal strength and vision, is assigned to chromosome region 11q12-13. Am J Hum Genet (1996) 59(1):146–51. PMC1915094 8659519

[155] Gong Y Slee RB Fukai N Rawadi G Roman-Roman S Reginato AM LDL receptor-related protein 5 (LRP5) affects bone accrual and eye development. Cell (2001) 107:513–23. 10.1016/s0092-8674(01)00571-2 11719191

[156] Ai M Heeger S Bartels CF Schelling DK Clinical and molecular findings in osteoporosis-pseudoglioma syndrome. Am J Hum Genet (2005) 77(5):741–53. 10.1086/497706 PMC1271384 16252235

[157] Qin M Hayashi H Oshima K Tahira T Hayashi K Kondo H Complexity of the genotype-phenotype correlation in familial exudative vitreoretinopathy with mutations in the LRP5 and/or FZD4 genes. Hum Mutat (2005) 26(2):104–12. 10.1002/humu.20191 15981244

[158] Patel MS Karsenty G Regulation of bone formation and vision by LRP5. N Engl J Med (2002) 346:1572–4. 10.1056/NEJM200205163462011 12015398

[159] Gregson CL Wheeler L Hardcastle SA Appleton LH Addison KA Brugmans M Mutations in Known Monogenic High Bone Mass Loci Only Explain a Small Proportion of High Bone Mass Cases. J Bone Miner Res (2015) 31(3):640–9. 10.1002/jbmr.2706 PMC4832273 26348019

[160] Roetzer KM Uyanik G Brehm A Zwerina J Zandieh S Czech T Novel familial mutation of LRP5 causing high bone mass: Genetic analysis, clinical presentation, and characterization of bone matrix mineralization. Bone (2018) 107:154–60. 10.1016/j.bone.2017.12.002 29208525

[161] Pangrazio A Boudin E Piters E Damante G Iacono N D'Elia AV Identification of the first deletion in the LRP5 gene in a patient with Autosomal Dominant Osteopetrosis type I. Bone (2011) 49(3):568–71. 10.1016/j.bone.2011.05.006 PMC3149657 21600326

[162] Costantini A Kekäläinen P Mäkitie RE Mäkitie O High bone mass due to novel LRP5 and AMER1 mutations. Eur J Med Genet (2017) 60(12):675–9. 10.1016/j.ejmg.2017.09.001 28893644

[163] Ai M Holmen SL Van HW Williams BO Warman ML Reduced affinity to and inhibition by DKK1 form a common mechanism by which high bone mass-associated missense mutations in LRP5 affect canonical Wnt signaling. Mol Cell Biol (2005) 25(12):4946–55. 10.1128/MCB.25.12.4946-4955.2005 PMC1140571 15923613

[164] Balemans W Piters E Cleiren E Ai M Van Wesenbeeck L Warman ML The binding between sclerostin and LRP5 is altered by DKK1 and by high-bone mass LRP5 mutations. Calcif Tissue Int (2008) 82(6):445–53. 10.1007/s00223-008-9130-9 18521528

[165] Pekkinen M Grigelioniene G Akin L Shah K Karaer K Kurtoğlu S Novel mutations in the LRP5 gene in patients with Osteoporosis-pseudoglioma syndrome. Am J Med Genet A (2017) 173(12):3132–5. 10.1002/ajmg.a.38491 29055141

[166] Alonso N Soares DC McCloskey EV Summers GD Ralston SH Gregson CL Atypical Femoral Fracture in Osteoporosis Pseudoglioma Syndrome Associated with Two Novel Compound Heterozygous Mutations in LRP5. J Bone Min Res (2015) 30(4):615–20. 10.1002/jbmr.2403 25384351

[167] Moon RT Kohn AD Ferrari GVD Kaykas A WNT and [beta]-catenin signalling: diseases and therapies. Nat Rev Genet (2004) 5(9):691–701. 10.1038/nrg1427 15372092

[168] Clevers H Wnt/β-Catenin Signaling in Development and Disease. Cell (2006) 127(3):469–80. 10.1016/j.cell.2006.10.018 17081971

[169] Babij P Zhao W Small C Kharode Y Yaworsky PJ Bouxsein ML High bone mass in mice expressing a mutant LRP5 gene. J Bone Miner Res (2003) 18(6):960–74. 10.1359/jbmr.2003.18.6.960 12817748

[170] Bueno M Oliván G Jiménez A Garagorri JM Sarría A Bueno AL Sclerosteosis in a Spanish male: first report in a person of Mediterranean origin. J Med Genet (1994) 31(12):976–7. 10.1136/jmg.31.12.976 PMC1016704 7891385

[171] Itin PH Keserü B Hauser V Syndactyly/Brachyphalangy and Nail Dysplasias as Marker Lesions for Sclerosteosis. Dermatology (2001) 202(3):259–60. 10.1159/000051649 11385236

[172] Butler WT Mikulski A Urist MR Bridges G Uyeno S Noncollagenous proteins of a rat dentin matrix possessing bone morphogenetic activity. J Dent Res (1977) 56(3):228–32. 10.1177/00220345770560030601 265954

[173] Tsukamoto S Mizuta T Fujimoto M Ohte S Osawa K Miyamoto A Smad9 is a new type of transcriptional regulator in bone morphogenetic protein signaling. Sci Rep (2014) 4:7596. 10.1038/srep07596 25534700 PMC4274517

[174] Sousa SB Jenkins D Chanudet E Tasseva G Ishida M Anderson G Gain-of-function mutations in the phosphatidylserine synthase 1 (PTDSS1) gene cause Lenz-Majewski syndrome. Nat Genet (2014) 46(1):70–6. 10.1038/ng.2829 24241535

[175] Gnepp DR Diagnostic surgical pathology of the head and neck. Philadelphia: Saunders (2001).

[176] Belsky JL Hamer JS Hubert JE Insogna K Johns W Torus Palatinus: A New Anatomical Correlation with Bone Density in Postmenopausal Women. J Clin Endo Metab (2003) 88(5):2081–6. 10.1210/jc.2002-021726 12727958

[177] Hosoi T Yoda T Yamaguchi M Amano H Orimo H Elderly women with oral exostoses had higher bone mineral density. J Bone Miner Metab (2003) 21(2):120–2. 10.1007/s007740300020 12601578

[178] Haapasalo H Kannus P Sievanen H Pasanen M Uusi-Rasi K Heinonen A Effect of long-term unilateral activity on bone mineral density of female junior tennis players. J Bone Miner Res (1998) 13(2):310–9. 10.1359/jbmr.1998.13.2.310 9495526

[179] Gregson CL Paggiosi MA Crabtree N Steel SA McCloskey E Duncan EL Analysis of body composition in individuals with high bone mass reveals a marked increase in fat mass in women but not men. J Clin Endocrinol Metab (2013) 98(2):818–28. 10.1210/jc.2012-3342 PMC3589712 23337721

[180] Nevitt MC Lane NE Scott JC Hochberg MC Pressman AR Genant HK Radiographic osteoarthritis of the hip and bone mineral density. The Study of Osteoporotic Fractures Research Group. Arthritis Rheum (1995) 38(7):907–16. 10.1002/art.1780380706 7612040

[181] Burger H van Daele PL Odding E Valkenburg HA Hofman A Grobbee DE Association of radiographically evident osteoarthritis with higher bone mineral density and increased bone loss with age. The Rotterdam Study. Arthritis Rheum (1996) 39(1):81–6. 10.1002/art.1780390111 8546742

[182] Chaganti RK Parimi N Lang T Orwoll E Stefanick ML Nevitt M Bone mineral density and prevalent osteoarthritis of the hip in older men for the Osteoporotic Fractures in Men (MrOS) Study Group. Osteoporos Int (2010) 21(8):1307–16. 10.1007/s00198-009-1105-9 PMC3354730 20101493

[183] Antoniades L MacGregor AJ Matson M Spector TD A cotwin control study of the relationship between hip osteoarthritis and bone mineral density. Arthritis Rheumatism (2000) 43(7):1450–5. 10.1002/1529-0131(200007)43:7<1450::AID-ANR6>3.0.CO;2-6 10902745

[184] Hardcastle SA Gregson CL Deere KC Davey SG Dieppe P Tobias JH High bone mass is associated with an increased prevalence of joint replacement: a case-control study. Rheumatol (Oxford) (2013) 52(6):1042–51. 10.1093/rheumatology/kes411 PMC3651613 23362220

[185] Hardcastle SA Dieppe P Gregson CL Hunter D Thomas GE Arden NK Prevalence of radiographic hip osteoarthritis is increased in high bone mass. Osteoarthritis Cartilage (2014) 22(8):1120–8. 10.1016/j.joca.2014.06.007 PMC4147962 24971870

[186] Hardcastle SA Dieppe P Gregson CL Arden NK Spector TD Hart DJ Individuals with high bone mass have an increased prevalence of radiographic knee osteoarthritis. Bone (2015) 71:171–9. 10.1016/j.bone.2014.10.015 PMC4289915 25445455

[187] Gregson CL Hardcastle SA Murphy A Faber B Fraser WD Williams M High Bone Mass is associated with bone-forming features of osteoarthritis in non-weight bearing joints independent of body mass index. Bone (2017) 97:306–13. 10.1016/j.bone.2017.01.005 PMC5378151 28082078

[188] Hardcastle SA Dieppe P Gregson CL Arden NK Spector TD Hart DJ Osteophytes, enthesophytes and High Bone Mass; A bone-forming triad with relevance for osteoarthritis? Arthritis Rheum (2014) 66(9):2429–39. 10.1002/art.38729 PMC4288267 24910132

[189] Hartley A Hardcastle SA Paternoster L McCloskey E Poole KES Javaid MK Individuals with High Bone Mass have increased progression of radiographic and clinical features of knee osteoarthritis. Osteoarthritis Cartilage (2020) 28(9)1180-90. 10.1016/j.joca.2020.03.020 32417557

[190] Tolar J Teitelbaum SL Orchard PJ Osteopetrosis. N Eng J Med (2004) 351(27):2839–49. 10.1056/NEJMra040952 15625335

[191] Albers-Schonberg HE Rontgenbilder einer seltenen Knockenerkrankung. Munch Med Wochenschr (1903) 5:365–8.

[192] Whyte MP Sclerosing Bone Disorders. In: Rosen CJ, editor. Primer on the Metabolic Bone Diseases and Disorders of Mineral Metabolism: American Society for Bone and Mineral Research. Washington, USA: The Sheridan Press (2008). p. 412–23.

[193] Balemans W Van WL Van HW A clinical and molecular overview of the human osteopetroses. CalcifTissue Int (2005) 77(5):263–74. 10.1007/s00223-005-0027-6 16307387

[194] Aker M Rouvinski A Hashavia S Ta-Shma A Shaag A Zenvirt S An SNX10 mutation causes malignant osteopetrosis of infancy. J Med Genet (2012) 49(4):221–6. 10.1136/jmedgenet-2011-100520 22499339

[195] Megarbane A Pangrazio A Villa A Chouery E Maarawi J Sabbagh S Homozygous stop mutation in the SNX10 gene in a consanguineous Iraqi boy with osteopetrosis and corpus callosum hypoplasia. Eur J Med Genet (2013) 56(1):32–5. 10.1016/j.ejmg.2012.10.010 23123320

[196] Sobacchi C Frattini A Guerrini MM Abinun M Pangrazio A Susani L Osteoclast-poor human osteopetrosis due to mutations in the gene encoding RANKL. Nat Genet (2007) 39(8):960–2. 10.1038/ng2076 17632511

[197] Segovia-Silvestre T Neutzsky-Wulff A Sorensen M Christiansen C Bollerslev J Karsdal M Advances in osteoclast biology resulting from the study of osteopetrotic mutations. Hum Genet (2009) 124(6):561–77. 10.1007/s00439-008-0583-8 18987890

[198] Del FA Fornari R Van WL de Freitas F Timmermans JP Peruzzi B A new heterozygous mutation (R714C) of the osteopetrosis gene, pleckstrin homolog domain containing family M (with run domain) member 1 (PLEKHM1), impairs vesicular acidification and increases TRACP secretion in osteoclasts. J Bone MinerRes (2008) 23(3):380–91. 10.1359/jbmr.071107 17997709

[199] Malinin NL Zhang L Choi J Ciocea A Razorenova O Ma YQ A point mutation in KINDLIN3 ablates activation of three integrin subfamilies in humans. Nat Med (2009) 15(3):313–8. 10.1038/nm.1917 PMC2857384 19234460

[200] Pasvolsky R Feigelson SW Kilic SS Simon AJ Tal-Lapidot G Grabovsky V A LAD-III syndrome is associated with defective expression of the Rap-1 activator CalDAG-GEFI in lymphocytes, neutrophils, and platelets. J Exp Med (2007) 204(7):1571–82. 10.1084/jem.20070058 PMC2118641 17576779

[201] Miryounesi M Nikfar A Changi-Ashtiani M Shahrooei M Dinmohammadi H Shahani T A novel homozygous LRRK1 stop gain mutation in a patient suspected with osteosclerotic metaphyseal dysplasia. Ann Hum Genet (2020) 84(1):102–6. 10.1111/ahg.12352 31571209

[202] Xing W Liu J Cheng S Vogel P Mohan S Brommage R Targeted disruption of leucine-rich repeat kinase 1 but not leucine-rich repeat kinase 2 in mice causes severe osteopetrosis. J Bone Miner Res (2013) 28(9):1962–74. 10.1002/jbmr.1935 PMC9528686 23526378

[203] Benichou O Cleiren E Gram J Bollerslev J de Vernejoul MC Van HW Mapping of autosomal dominant osteopetrosis type II (Albers-Schonberg disease) to chromosome 16p13.3. Am J Hum Genet (2001) 69(3):647–54. 10.1086/323132 PMC1235505 11468688

[204] Bollerslev J Mosekilde L Autosomal dominant osteopetrosis. Clin OrthopRelat Res (1993) 294):45–51. 10.1097/00003086-199309000-00006 8358946

[205] Waguespack SG Hui SL DiMeglio LA Econs MJ Autosomal Dominant Osteopetrosis: Clinical Severity and Natural History of 94 Subjects with a Chloride Channel 7 Gene Mutation. J Clin Endo Metab (2007) 92(3):771–8. 10.1210/jc.2006-1986 17164308

[206] Benichou OD Laredo JD de Vernejoul MC Type II autosomal dominant osteopetrosis (Albers-Schonberg disease): clinical and radiological manifestations in 42 patients. Bone (2000) 26(1):87–93. 10.1016/S8756-3282(99)00244-6 10617161

[207] Gelb BD Shi GP Chapman HA Desnick RJ Pycnodysostosis, a lysosomal disease caused by cathepsin K deficiency. Science (1996) 273:1236–8. 10.1126/science.273.5279.1236 8703060

[208] Donnarumma M Regis S Tappino B Rosano C Assereto S Corsolini F Molecular Analysis and Characterization of Nine Novel CTSK Mutations in Twelve Patients Affected by Pycnodysostosis. Mutation in brief# 961. Online. Hum Mutat (2007) 28:524. 10.1002/humu.9490 17397052

[209] Fujita Y Nakata K Yasui N Matsui Y Kataoka E Hiroshima K Novel Mutations of the Cathepsin K Gene in Patients with Pycnodysostosis and Their Characterization. J Clin Endo Metab (2000) 85:425–31. 10.1210/jcem.85.1.6247 10634420

[210] Hellemans J Preobrazhenska O Willaert A Debeer P Verdonk PC Costa T Loss-of-function mutations in LEMD3 result in osteopoikilosis, Buschke-Ollendorff syndrome and melorheostosis. Nat Genet (2004) 36(11):1213–8. 10.1038/ng1453 15489854

[211] Freyschmidt J Melorheostosis: a review of 23 cases. Eur Radiol (2001) 11(3):474–9. 10.1007/s003300000562 11288855

[212] Gass JK Hellemans J Mortier G Griffiths M Burrows NP Buschke-Ollendorff syndrome: a manifestation of a heterozygous nonsense mutation in the LEMD3 gene. J Am Acad Dermatol (2008) 58(5 Suppl 1):S103–S4. 10.1016/j.jaad.2007.03.031 18489034

[213] Kang H Jha S Ivovic A Fratzl-Zelman N Deng Z Mitra A Somatic SMAD3-activating mutations cause melorheostosis byup-regulating the TGF-β/SMAD pathway. J Exp Med (2020) 217(5):e20191499. 10.1084/jem.20191499 32232430 PMC7201932

[214] De Ridder R Boudin E Zillikens MC Ibrahim J van der Eerden BCJ Van Hul W A multi-omics approach expands the mutational spectrum of MAP2K1-related melorheostosis. Bone (2020) 137:115406. 10.1016/j.bone.2020.115406 32387835

[215] Jenkins ZA van Kogelenberg M Morgan T Aaron J Ryuji F Esther P Germline mutations in WTX cause a sclerosing skeletal dysplasia but do not predispose to tumorigenesis. Nat Genet (2009) 41(1):95–100. 10.1038/ng.270 19079258

[216] Lemire EG Wiebe S Clinical and radiologic findings in an adult male with dysosteosclerosis. Am J Med Genet A (2008) 146a(4):474–8. 10.1002/ajmg.a.32182 18203158

[217] Elcioglu NH Vellodi A Hall CM Dysosteosclerosis: a report of three new cases and evolution of the radiological findings. J Med Genet (2002) 39(8):603–7. 10.1136/jmg.39.8.603 PMC1735202 12161605

[218] Campeau PM Lu JT Sule G Jiang MM Bae Y Madan S Whole-exome sequencing identifies mutations in the nucleoside transporter gene SLC29A3 in dysosteosclerosis, a form of osteopetrosis. Hum Mol Genet (2012) 21(22):4904–9. 10.1093/hmg/dds326 PMC3607481 22875837

[219] Guo L Bertola DR Takanohashi A Saito A Segawa Y Yokota T Bi-allelic CSF1R Mutations Cause Skeletal Dysplasia of Dysosteosclerosis-Pyle Disease Spectrum and Degenerative Encephalopathy with Brain Malformation. Am J Hum Genet (2019) 104(5):925–35. 10.1016/j.ajhg.2019.03.004 PMC6507048 30982609

[220] Kinoshita A Saito T Tomita H Makita Y Yoshida K Ghadami M Domain-specific mutations in TGFB1 result in Camurati-Engelmann disease. Nat Genet (2000) 26(1):19–20. 10.1038/79128 10973241

[221] Campos-Xavier A Saraiva J Savarirayan R Verloes A Feingold J Faivre L Phenotypic variability at the TGF-B1 locus in Camurati-Engelmann disease. Hum Genet (2001) 109(6):653–8. 10.1007/s00439-001-0644-8 11810278

[222] Smith R Walton RJ Corner BD Gordon IRS Clinical and Biochemical Studies in Engelmann’s Disease (Progressive Diaphyseal Dysplasia). QJM (1977) 46(2):273–94. 866579

[223] Crisp AJ Brenton DP Engelmann’s disease of bone–a systemic disorder? Ann Rheum Dis (1982) 41(2):183–8. 10.1136/ard.41.2.183 PMC1000905 7073346

[224] Saito T Kinoshita A Yoshiura K Makita Y Wakui K Honke K Domain-specific Mutations of a Transforming Growth Factor (TGF)-B1 Latency-associated Peptide Cause Camurati-Engelmann Disease Because of the Formation of a Constitutively Active Form of TGFB1. J Biol Chem (2001) 276(15):11469–72. 10.1074/jbc.C000859200 11278244

[225] McGowan NWA MacPherson H Janssens K Van Hul W Frith JC Fraser WD A Mutation Affecting the Latency-Associated Peptide of TGFB1 in Camurati-Engelmann Disease Enhances Osteoclast Formation in Vitro. J Clin Endo Metab (2003) 88(7):3321–6. 10.1210/jc.2002-020564 12843182

[226] Van Hul W Boudin E Vanhoenacker FM Mortier G Camurati-Engelmann Disease. Calcif Tissue Int (2019) 104(5):554–60. 10.1007/s00223-019-00532-1 30721323

[227] Ghosal SP Mukherjee AK Mukherjee D Ghosh AK Diaphyseal dysplasia associated with anemia. J Pediatr (1988) 113(1 Pt 1):49–57. 10.1016/S0022-3476(88)80527-4 3385529

[228] Genevieve D Proulle V Isidor B Bellais S Serre V Djouadi F Thromboxane synthase mutations in an increased bone density disorder (Ghosal syndrome). Nat Genet (2008) 40(3):284–6. 10.1038/ng.2007.66 18264100

[229] Jagtap R Alansari R Ruprecht A Kashtwari D Trichodentoosseous syndrome: a case report and review of literature. BJR Case Rep (2019) 5(4):20190039. 10.1259/bjrcr.20190039 31938567 PMC6945255

[230] Whyte MP Hughes AE Expansile skeletal hyperphosphatasia is caused by a 15-base pair tandem duplication in TNFRSF11A encoding RANK and is allelic to familial expansile osteolysis. J Bone Min Res (2002) 17(1):26–9. 10.1359/jbmr.2002.17.1.26 11771666

[231] Nakatsuka K Nishizawa Y Ralston SH Phenotypic characterization of early onset Paget’s disease of bone caused by a 27-bp duplication in the TNFRSF11A gene. J Bone MinerRes (2003) 18(8):1381–5. 10.1359/jbmr.2003.18.8.1381 12929927

[232] Whyte MP Murphy WA Siegel BA 99mTc-pyrophosphate bone imaging in osteopoikilosis, osteopathia striata, and melorheostosis. Radiology (1978) 127(2):439–43. 10.1148/127.2.439 205901

[233] Benichou OD Benichou B Copin H de Vernejoul MC Van HW Further evidence for genetic heterogeneity within type II autosomal dominant osteopetrosis. J Bone Miner Res (2000) 15(10):1900–4. 10.1359/jbmr.2000.15.10.1900 11028441

[234] Maroteaux P Lamy M La Pycnodysostose. Presse Med (1962) 70:999–1002. 14470123

[235] Maroteaux P Lamy M The Malady of Toulouse-Lautrec. JAMA (1965) 191:715–7. 10.1001/jama.1965.03080090029007 14245511

[236] Bartsocas CS Pycnodysostosis: Toulouse-Lautrec’s and Aesop’s disease? Hormones (Athens) (2002) 1(4):260–2. 10.14310/horm.2002.1177 17018457

[237] Bonafe L Cormier-Daire V Hall C Lachman R Mortier G Mundlos S Nosology and classification of genetic skeletal disorders: 2015 revision. Am J Med Genet A (2015) 167a(12):2869–92. 10.1002/ajmg.a.37365 26394607

[238] Kornak U Kasper D Bosl MR Kaiser E Schweizer M Schulz A Loss of the ClC-7 chloride channel leads to osteopetrosis in mice and man. Cell (2001) 104(2):205–15. 10.1016/S0092-8674(01)00206-9 11207362

[239] Zhang ZL He JW Zhang H Hu WW Fu WZ Gu JM Identification of the CLCN7 gene mutations in two Chinese families with autosomal dominant osteopetrosis (type II). J Bone Min Metab (2009) 27(4):444–51. 10.1007/s00774-009-0051-0 19288050

[240] Pangrazio A Pusch M Caldana E Frattini A Lanino E Tamhankar PM Molecular and clinical heterogeneity in CLCN7-dependent osteopetrosis: report of 20 novel mutations. Hum Mutat (2010) 31(1):E1071–E80. 10.1002/humu.21167 19953639

[241] Waguespack SG Koller DL White KE Fishburn T Carn G Buckwalter KA Chloride Channel 7 (ClCN7) Gene Mutations and Autosomal Dominant Osteopetrosis, Type II. J Bone Min Res (2003) 18(8):1513–8. 10.1359/jbmr.2003.18.8.1513 12929941

[242] Bollerslev J Osteopetrosis. A genetic and epidemiological study. Clin Genet (1987) 31(2):86–90. 10.1111/j.1399-0004.1987.tb02774.x 3829443

[243] Salzano FM Osteopetrosis: review of dominant cases and frequency in a Brazilian state. Acta Genet Med Gemellol (Roma) (1961) 10:353–8. 10.1017/S1120962300016954 14496532

[244] VanHul W Bollerslev J Gram J VanHul E Wuyts W Benichou O Localization of a gene for autosomal dominant osteopetrosis (Albers-Schonberg disease) to chromosome 1p21. Am J Hum Genet (1997) 61(2):363–9. 10.1086/514844 PMC1715917 9311741

[245] Motyckova G Fisher DE Pycnodysostosis: role and regulation of cathepsin K in osteoclast function and human disease. Curr Mol Med (2002) 2(5):407–21. 10.2174/1566524023362401 12125807

[246] Muto T Michiya H Taira H Murase H Kanazawa M Pycnodysostosis. Report of a case and review of the Japanese literature, with emphasis on oral and maxillofacial findings. Oral Surg Oral Med Oral Pathol (1991) 72(4):449–55. 10.1016/0030-4220(91)90559-U 1923445

[247] Yates CJ Bartlett MJ Ebeling PR An atypical subtrochanteric femoral fracture from pycnodysostosis: A lesson from nature. J Bone Min Res (2011) 26(6):1377–9. 10.1002/jbmr.308 21611976

[248] Eisman JA Bone HG Hosking DJ McClung MR Reid IR Rizzoli R Odanacatib in the treatment of postmenopausal women with low bone mineral density: Three-year continued therapy and resolution of effect. J Bone Min Res (2011) 26(2):242–51. 10.1002/jbmr.212 20740685

[249] McHugh KP Hodivala-Dilke K Zheng MH Namba N Lam J Novack D Mice lacking beta3 integrins are osteosclerotic because of dysfunctional osteoclasts. J Clin Invest (2000) 105(4):433–40. 10.1172/JCI8905 PMC289172 10683372

[250] Morgan EA Schneider JG Baroni TE Uluckan O Heller E Hurchla MA Dissection of platelet and myeloid cell defects by conditional targeting of the Beta 3-integrin subunit. FASEB J (2010) 24(4):1117–27. 10.1096/fj.09-138420 PMC2845430 19933310

[251] Peretz H Rosenberg N Landau M Usher S Nelson EJR Mor-Cohen R Molecular diversity of Glanzmann thrombasthenia in southern India: new insights into mRNA splicing and structure-function correlations of aIIbB3 integrin (ITGA2B, ITGB3). Hum Mutat (2006) 27(4):359–69. 10.1002/humu.20304 16463284

[252] Yarali N Fisgin T Duru F Kara A Osteopetrosis and Glanzmann’s thrombasthenia in a child. Ann Hematol (2003) 82(4):254–6. 10.1007/s00277-002-0571-3 12707732

[253] Feng X Novack DV Faccio R Ory DS Aya K Boyer MI A Glanzmann’s mutation in beta 3 integrin specifically impairs osteoclast function. J Clin Invest (2001) 107(9):1137–44. 10.1172/JCI12040 PMC209281 11342577

[254] Goltz RW Peterson WC Gorlin RJ Ravits HG Focal Dermal Hypoplasia. Arch Dermatol (1962) 86(6):708–17. 10.1001/archderm.1962.01590120006002 13948891

[255] Goltz RW Focal Dermal Hypoplasia Syndrome: An Update. Arch Dermatol (1992) 128(8):1108–11. 10.1001/archderm.128.8.1108 1497368

[256] Wang X Reid Sutton V Omar Peraza-Llanes J Yu Z Rosetta R Kou YC Mutations in X-linked PORCN, a putative regulator of Wnt signaling, cause focal dermal hypoplasia. Nat Genet (2007) 39(7):836–8. 10.1038/ng2057 17546030

[257] Grafe I Yang T Alexander S Homan EP Lietman C Jiang MM Excessive transforming growth factor-β signaling is a common mechanism in osteogenesis imperfecta. Nat Med (2014) 20(6):670–5. 10.1038/nm.3544 PMC4048326 24793237

[258] Oliveri MB Cassinelli H Bergadá C Mautalen CA Bone mineral density of the spine and radius shaft in children with X-linked hypophosphatemic rickets (XLH). Bone Miner (1991) 12(2):91–100. 10.1016/0169-6009(91)90038-2 2015415

[259] Skrinar A Dvorak-Ewell M Evins A Macica C Linglart A Imel EA The Lifelong Impact of X-Linked Hypophosphatemia: Results From a Burden of Disease Survey. J Endocrine Soc (2019) 3(7):1321–34. 10.1210/js.2018-00365 PMC6595532 31259293

[260] Gensure RC Mäkitie O Barclay C Chan C Depalma SR Bastepe M A novel COL1A1 mutation in infantile cortical hyperostosis (Caffey disease) expands the spectrum of collagen-related disorders. J Clin Invest (2005) 115(5):1250–7. 10.1172/JCI22760 PMC1087158 15864348

[261] Letko A Leuthard F Jagannathan V Corlazzoli D Matiasek K Schweizer D Whole Genome Sequencing Indicates Heterogeneity of HyperostoticDisorders in Dogs. Genes (2020) 11(2):163. 10.3390/genes11020163 PMC7074049 32033218

[262] Plomin R Haworth CM Davis OS Common disorders are quantitative traits. Nat Rev Genet (2009)10(12):872–8. 10.1038/nrg2670 19859063

[263] Lanktree MB Hegele RA Schork NJ Spence JD Extremes of unexplained variation as a phenotype: an efficientapproach for genome-wide association studies of cardiovascular disease. CircCardiovasc Genet (2010) 3(2):215–21. 10.1161/CIRCGENETICS.109.934505 PMC3084495 20407100

[264] Robinson EB Koenen KC McCormick MC Munir K Hallett V Happe F Evidence that autistic traits show the same etiology in the generalpopulation and at the quantitative extremes (5%, 2.5%, and 1%). Arch GenPsychiatry (2011)68(11):1113–21. 10.1001/archgenpsychiatry.2011.119 PMC3708488 22065527

[265] Hu S Zhong Y Hao Y Luo M Zhou Y Guo H Novel rare alleles of ABCA1 are exclusively associated with extremehigh-density lipoprotein-cholesterol levels among the Han Chinese. Clin ChemLab Med (2009) 47(10):1239–45. 10.1515/CCLM.2009.284 19743957

[266] Paternoster L Evans DM Nohr EA Holst C Gaborieau V Brennan P Genome-wide population-based association study of extremely overweight young adults–the GOYA study. PLoS One (2011) 6(9):e24303. 10.1371/journal.pone.0024303 21935397 PMC3174168

[267] Barnett IJ Lee S Lin X Detecting rare variant effects using extreme phenotype sampling in sequencing association studies. Genet Epidemiol (2013) 37(2):142–51. 10.1002/gepi.21699 PMC3601902 23184518

[268] Rivadeneira F Styrkársdottir U Estrada K Halldórsson BV Hsu YH Richards JB Twenty bone-mineral-density loci identified by large-scale meta-analysis of genome-wide association studies. Nat Genet (2009) 41(11):1199–206. 10.1038/ng.446 PMC2783489 19801982

[269] Jaubert J Jaubert F Martin N Lee BK Eicher EM Guenet JL Three new allelic mouse mutations that cause skeletal overgrowth involve the natriuretic peptide receptor C gene (Npr3). Proc Natl Acad Sci U S A (1999) 96(18):10278–83. 10.1073/pnas.96.18.10278 PMC17879 10468599

[270] Esapa CT Piret SE Nesbit MA Loh NY Thomas G Croucher PI Mice with an N-Ethyl-N-Nitrosourea (ENU) Induced Tyr209Asn Mutation in Natriuretic Peptide Receptor 3 (NPR3) Provide a Model for Kyphosis Associated with Activation of the MAPK Signaling Pathway. PLoS One (2016) 11(12):e0167916. 10.1371/journal.pone.0167916 27959934 PMC5154531

[271] Matsukawa N Grzesik WJ Takahashi N Pandey KN Pang S Yamauchi M The natriuretic peptide clearance receptor locally modulates the physiological effects of the natriuretic peptide system. Proc Natl Acad Sci U S A (1999) 96(13):7403–8. 10.1073/pnas.96.13.7403 PMC22098 10377427

[272] Beutler B, MUTAGENETIX (TM), B. Beutler and colleagues Center for the Genetics of Host Defense. UT Southwestern, Dallas, TX Available at: https://mutagenetix.utsouthwestern.edu.

[273] Javed A Bae JS Afzal F Gutierrez S Pratap J Zaidi SK Structural coupling of Smad and Runx2 for execution of the BMP2 osteogenic signal. J Biol Chem (2008) 283(13):8412–22. 10.1074/jbc.M705578200 PMC2417186 18204048

[274] Palmer GD Attur MG Yang Q Liu J Moon P Beier F F-spondin deficient mice have a high bone mass phenotype. PLoS One (2014) 9(5):e98388. 10.1371/journal.pone.0098388 24875054 PMC4038615

[275] Appelman-Dijkstra NM Papapoulos SE Sclerostin Inhibition in the Management of Osteoporosis. Calc Tiss Int (2016) 98(4):370–80. 10.1007/s00223-016-0126-6 PMC4824823 27016922

[276] Nelson MR Tipney H Painter JL Shen J Nicoletti P Shen Y The support of human genetic evidence for approved drug indications. Nat Genet (2015) 47(8):856–60. 10.1038/ng.3314 26121088

[277] Recker RR Benson CT Matsumoto T Bolognese MA Robins DA Alam J A Randomized, Double-Blind Phase 2 Clinical Trial of Blosozumab, a Sclerostin Antibody, in Postmenopausal Women with Low Bone Mineral Density. J Bone Miner Res (2015) 30(2):216–24. 10.1002/jbmr.2351 25196993

[278] Glantschnig H Hampton R Wei N Scott K Nantermet P Zhao J Fully Human anti-DKK1 Antibodies Increase Bone Formation and Resolve Osteopenia in Mouse Models of Estrogen-Deficiency Induced Bone Loss. J Bone Miner Res (2008) 23:S60–S1.

[279] Heiland GR Zwerina K Baum W Kireva T Distler JAH Grisanti M Neutralisation of Dkk-1 protects from systemic bone loss during inflammation and reduces sclerostin expression. Ann Rheumat Dis (2010) 69(12):2152–9. 10.1136/ard.2010.132852 20858621

[280] Kulkarni NH Onyia JE Zeng Q Tian X Liu M Halladay DL Orally Bioavailable GSK-3a/b Dual Inhibitor Increases Markers of Cellular Differentiation In Vitro and Bone Mass In Vivo. J Bone Min Res (2006) 21(6):910–20. 10.1359/jbmr.060316 16753022

[281] Moore WJ Kern JC Bhat R Commons TJ Fukayama S Goljer I Modulation of Wnt Signaling Through Inhibition of Secreted Frizzled-Related Protein I (sFRP-1) with N-Substituted Piperidinyl Diphenylsulfonyl Sulfonamides. J Med Chem (2008) 52(1):105–16. 10.1021/jm801144h 19072540

[282] Harvey W The works of William Harvey. Philadelphia: University of Pennsylvania Press (1989).

